# Stuttering: the nature of the speech disruptions—a multimodal study of articulation and phonation

**DOI:** 10.3389/fnhum.2025.1623308

**Published:** 2025-09-09

**Authors:** Per A. Alm, Therése Brösemyr, Sofie Grinde, Sara Johansson, Jessica Karlsson, Gustav Nordgren, Ronja Olsson, Frida Rocksten, Maria Sandsten, Ingrid Sör, Denise White

**Affiliations:** ^1^Speech and Language Pathology, Department of Public Health and Caring Sciences, Uppsala University, Uppsala, Sweden; ^2^Centre for Mathematical Sciences, Lund University, Lund, Sweden

**Keywords:** stuttering, dystonia, Parkinson's disease, tremor, electromyography, larynx, vocal tract, speech motor control

## Abstract

Stuttering is a disorder characterized by transient disruptions in speech motor production. This article is focused on the characteristics of stuttering and the immediate vocal tract mechanisms resulting in stuttered speech disruptions. A range of observations from an initial series of studies on the motor characteristics of stuttering events in adults is presented, combined with a narrative review of published data regarding objective and subjective aspects of instances of stuttering. The aims of the empirical studies were to develop methods for data collection and analysis, as well as collecting and analyzing initial data. The analysis was exploratory and qualitative, focusing on physiological data from individual stuttering events in order to understand their underlying dynamics and mechanisms. As a frame of reference, the motor characteristics and subjective experiences of stuttering were compared with the characteristics of known movement disorders, such as dystonia, motor blocks (e.g., freezing of gait), and tremor. The results show that stuttering events can include both *negative* and *positive* motor signs. It is proposed that stuttered disruptions can arise both as a result of insufficient muscular activation and as a result of interfering dysfunctional muscular activity. It is further suggested that the characteristics of stuttering to a very high degree correspond to *motor block symptoms*, indicating a transient inability to execute the next motor program in the speech sequence. Elements of dystonia may account for some symptoms. Volitional attempts to break fixed postures may increase the muscular tension and result in tremor, similar to *dystonic tremor*. The severity of the tremor is strongly correlated with the severity of physical concomitants. Tremor may be silent, as well as resulting in rapid audible repetitions if the vocal tract is opened and closed at the tremor frequency. Silent periods in stuttering can result from total closure of the airway at the level of the larynx, the tongue, or the lips. However, silent periods can also result from excessive opening of the vocal folds, leading to silent airflow and an inability to phonate. It is proposed that the motor blocks stem from transient decoupling between cortical and basal ganglia networks.

## 1 Introduction

### 1.1 Background and aims

In 1957, Robert West, one of the founding figures in the field of speech-language pathology, stated that “Everyone but the expert knows what stuttering is” (West et al., 1957, p. 15). This statement should probably be viewed as an acknowledgment of the complexity of this speech disorder. It is unsurprising that the symptoms of stuttering have led to a multitude of interpretations and speculations. The scientific quest for an explanation has resulted in a wide range of theories addressing various aspects of stuttering, including motor control, sensory-motor integration, auditory feedback, language, cognition, emotions, anticipation, etc.

It seems fair to say that the characteristics and nature of the speech motor symptoms in stuttering are not yet well-understood. This article focuses on detailed analysis of the objective motor behaviors underlying speech disruptions in stuttering, building on the work of Conture et al. (1977), Freeman and Ushijima (1978), Shapiro (1980), Smith et al. (1996), and others. In neurology, such an approach is referred to as *phenomenology*—the description and classification of movement disorders based on observable motor signs and, where relevant, the patient's subjective experiences (Frucht and Termsarasab, 2020). In this article, the subjective experiences during moments of stuttering are discussed based on reports in the literature, while the focus of data analysis is the motor timeline of disruptions: What are the earliest detectable motor signs of a disfluency, and how does the breakdown unfold in real time? In essence, the phenomenology of stuttering (its audible and visible features) serves as a window into the malfunctions of the speech motor system. By analyzing these motor signs, we may infer clues for the immediate mechanisms—or “proximal causes”—of the disruptions. The proximal cause is the last step in a causal chain, with more distal causes within the realms of neurology, genetics, etc. A deeper understanding of the mechanisms can be expected to be informative for clinical interventions as well as for how stuttering is viewed—by the people who stutter and by society at large.

The introduction will begin with a review of known movement disorders with characteristics that overlap with stuttering, in order to provide a conceptual framework. The review of characteristics includes overt symptoms, subjective experiences, and known causal mechanisms. This will be followed by a review of existing research on stuttering regarding these aspects, with comparisons with the other disorders. Thereafter, results from three preliminary studies at Uppsala University will be presented. The overall aims of these studies were (1) to develop the methodologies for this type of research and (2) to acquire data for exploratory qualitative analysis of the motor characteristics of speech disruptions in stuttering.

### 1.2 Immediate mechanisms of stuttering: central vs. peripheral

What, specifically, goes wrong in the speech process when stuttering occurs? Numerous theories attempt to explain the nature of these breakdowns. In short, it seems that theories of the disruptions in stuttering may be boiled down to four main mechanisms: (1) That speech is interrupted by some control mechanisms in the brain, because it detects a real or an imagined speech error; (2) that the speech disruptions result from some type of interruption of the brain's speech processing, due to emotions, stress, motor dysregulation, unstable feedback control, etc.; (3) that the interruption occurs as a result of “mechanical” events within the vocal tract—in other words, that the actual disruption occurs in the periphery, not in the brain, and (4) a more or less unconcious intention to inhibit the attempt to talk. As an example of “peripheral disruptions,” excessive muscular tension might block the airflow, making it impossible to continue. A straightforward example of this type of theory was proposed by Starkweather (1995), stating that the proximal cause of stuttering is simply the elevated activity of the speech muscles. This view is also part of the proposals by Guitar (2024), who emphasizes the role of muscular tension triggered by stress as an important factor in stuttering.

### 1.3 Categorization of stuttering events

There have been many different attempts to categorize the core behaviors of stuttering (e.g., see Yairi and Seery, 2023), but the most established categorization appears to be a division into repetitions, prolongations, and blocks (Van Riper, 1982; Guitar, 2024). However, a limitation is that these categories are based on the listener's perspective, and therefore offer limited insight into the actual nature of the disruptions. Another problem is that stuttering events in reality often display a combination of repetitions, prolongations, and blocks, within the same event.

A more recent approach, the Lidcombe Behavioral Data Language (Teesson et al., 2003), classifies stuttering based on speech movements rather than auditory perception. It identifies three main categories: (1) repeated movements, (2) fixed postures, and (3) superfluous behaviors. This system provides a physiologically relevant description of stuttering events, even though it is limited in detail.

### 1.4 Neurological motor conditions overlapping with stuttering

#### 1.4.1 Motor block symptoms, dystonia, and tremor

Exploring parallels between stuttering and known neurological movement disorders may clarify the nature and mechanisms of stuttering and refine its definition. Based on the symptoms of stuttering, three neurological motor conditions may be particularly relevant: (1) *motor blocks* (e.g., freezing of gait), (2) *dystonia*, and (3) *tremor*. These conditions will be presented in the following section, to be followed by a discussion of specific aspects of the disorders in relation to stuttering. These aspects are: (1) *Task-specificity and variability of motor symptoms*, (2) *loss of volitional control and the sense of being “stuck,”* (3) *reactions to motor dysfunctions*, and (4) *brain findings and pharmacology*.

##### 1.4.1.1 Motor block disorders

The most well-known type of motor block symptom is freezing of gait (FOG) in Parkinson's disease, but similar motor blocks are reported in other activities, such as hand movements, speech (Vercruysse et al., 2014), and swallowing (Labeit et al., 2020). The term “motor block” was proposed by Giladi et al. (1992) as a general term for episodic motor phenomena similar to FOG, irrespective of the effectors involved. FOG involves sudden episodes where patients cannot move their feet forward despite the intention to walk, often described as feet being “glued” to the ground. Electromyography (EMG) has shown co-contraction of lower leg muscles with slightly elevated tonus—on average about 20% higher than the tonus when walking (Günther et al., 2019). These episodes, lasting from brief moments to over 30 s, often occur when initiating walking or when altering the walking pattern (Nutt et al., 2011). Vercruysse et al. (2014) noted that it is sequential movements, such as walking, writing, or speech, that are affected by FOG-like symptoms.

It is interesting to note that the term “motor block” corresponds to the original use of the term “block” in stuttering by Charles Van Riper. He described it as a temporary inability to move the speech musculature: “the stutterer finds himself unable to move a certain speech structure when it is necessary for him to do” (Van Riper, 1939, pp. 324–325). This meaning differs from the typical meaning of blocks in later literature on stuttering, implying a block of the airflow (e.g., Van Riper, 1982, p. 121).

##### 1.4.1.2 Dystonia

Dystonia is a condition characterized by dysregulated muscle tone, causing tense involuntary contractions. It can occur as a primary disorder or as a symptom of other conditions, such as Parkinson's disease or medication side effects. Kiziltan and Akalin (1996) suggested that the involuntary movements in stuttering resemble those in dystonic syndromes, proposing that stuttering may represent a form of action dystonia. These similarities were further explored in Alm (2004).

##### 1.4.1.3 Tremor

Tremor is characterized by involuntary, rhythmic muscle contractions, often described as *oscillations*. Tremor can occur as a primary disorder or as a symptom in other movement disorders. Mild tremor tendencies are normal, i.e., physiological tremor. Exaggerated tremor in speech-related muscles sometimes occurs in stuttering (e.g., Fibiger, 1971; Smith et al., 1993).

#### 1.4.2 Variability and task-specificity

##### 1.4.2.1 Stuttering

Stuttering primarily manifests as motor anomalies that are limited to the intention of speaking, particularly for the purpose of communication. This means that stuttering is *task-specific*. In addition, the motor symptoms of stuttering often show great *variability*, between situations and from one moment to the next. Altering the way of speaking or the speaking conditions can significantly reduce stuttering, temporarily—such as imitating an accent, whispering, speaking with rhythm, choral reading, manipulation of the auditory feedback, or talking alone with no intent to communicate (Bloodstein et al., 2021; Jackson et al., 2021). Similarly, internal changes may also temporarily increase fluency. Examples include states of strong anger, enthusiasm, or fear, but also change of the focus of attention (Alm, 2014; Bloodstein et al., 2021).

##### 1.4.2.2 Motor block symptoms

Motor block symptoms, such as FOG in Parkinson's disease, also exhibit task-specificity and influence of the emotional and attentional states. For example, patients with FOG may run or climb stairs more easily than walking on flat ground (Frucht and Termsarasab, 2020, p. 206). Rhythmic cues and visual targets when walking can have an alleviating effect (Nutt et al., 2011), as well as heightened emotions (Fahn, 1995). Episodes of FOG often occur during movement initiation or changes in the movements, such as turning or approaching a chair (Fahn, 1995)—suggesting that the shifting to a new motor program may be the core problem. The symptoms are exacerbated by time pressure. Patients often use “trick maneuvers” (e.g., jerky head movements or novel walking patterns) to overcome FOG. However, it is reported that such maneuvers tend to be inconsistently effective (Fahn, 1995), similarly to such maneuvers in stuttering.

##### 1.4.2.3 Dystonia

Frucht and Termsarasab (2020, pp. 142–143) listed three characteristic features suggesting the diagnosis of dystonia. Two of these features are related to variability of the symptoms: task-specificity and “sensory trick.”

*Task-specificity*. Task-specific dystonia is a diagnosis of involuntary contractions that typically affect highly automatized tasks requiring complex sequential movements, such as writing or playing musical instruments (Sadnicka et al., 2018). Frucht and Termsarasab (2020) pointed out that dystonia that traditionally is not labeled “task-specific” also tend to show task-specificity. For example, patients with blepharospasm may report that the eye closes only when they speak or when they read. Patients with leg dystonia may notice that the contractions are triggered specifically by walking upstairs, downstairs, or by running. In laryngeal dystonia, specific words or speech sounds may trigger the problems (Blitzer et al., 2018).

*Sensory trick* means that the dystonia is ameliorated by some type of sensory stimulation. For instance, blepharospasm might be relieved by touching the forehead or cervical dystonia reduced by touching the cheek. Sensory effects also occur in stuttering. The most well-known is the effect of auditory manipulation, such as masking noise or delayed or frequency altered auditory feedback (Bloodstein et al., 2021). In addition, there are stuttering persons using somatosensory tricks, such as pinching their leg or keeping a lozenge under the tongue when talking (personal contact).

##### 1.4.2.4 Tremor

Task-specific tremor is a type of action tremor that shares many characteristics of task-specific dystonia, but the main motor symptom is trembling. Similarly to task-specific dystonia, it particularly affects skilled and well-learned tasks, such as writing, playing a musical instrument, or a golfer's putting (Bain, 2011). The nature of the relationship between task-specific tremor and task-specific dystonia is a matter of debate. In short, task-specific tremor is yet another example of how the complexity of the motor system can result in a multitude of various symptoms.

#### 1.4.3 Loss of volitional control and sense of getting stuck

In order to understand the nature of a motor disorder, it is of interest to also include the subjective experiences of the affected persons. Charles Van Riper and Wendell Johnson, two of the most influential figures in the field of stuttering during the 1900s, both stuttered themselves and collaborated at the University of Iowa in the 1930s. Van Riper (1992, p. 82) recalled an early discussion with Johnson about the core of stuttering:

“I recall that we finally agreed that the core was a sticking, an involuntary but very brief inability to move one or more parts of our speech mechanism.”

However, their agreement did not last. Van Riper later wrote to Johnson:

“You now seem to have given up your earlier belief that its core consists of a momentary involuntary blocking to which all the other learned abnormal behaviors have attached themselves. Am I correct in thinking that you now believe that the core of stuttering is a learned “disfluency?” If so, I fear I cannot share that view. Not disfluency but gluency^1^ is the essence of our disorder.... we get stuck when we stutter” (1992, p. 83)

Wendell Johnson did develop his new view, that the core of stuttering is learned and that stuttering can be described as something that one “does,” not something that “happens” (e.g., Johnson, 1961). Perkins (1990), however, followed the reasoning by Van Riper, and defined stuttering as an involuntary disruption of speech, where “involuntary” meant a temporary loss of conscious control. More recently, Tichenor and Yaruss (2018) interviewed adults about their experiences before, during and after moments of stuttering. A main result was the sense of loss of control, as exemplified by the following quotations (p. 1185):

“The loss of control feels like a blip in the system where there's like a brief instant where whatever plans that are occurring are interrupted.”

“... being in a moment of stuttering and knowing where you want your mouth, lips, and tongue to move, and knowing how you want it to sound, and literally not being able to produce that word.”

It is noteworthy that the experiences of loss of control reported by people who stutter are strikingly similar to those described by individuals with FOG, dystonia, or tremor. Specifically, individuals experiencing motor blocks, such as FOG, tend to describe their experiences using terms almost identical to those used by people who stutter, as quoted above. For example, people with FOG often describe the sensation as feeling like their feet are “glued to the floor” (Nutt et al., 2011).

In summary, subjective reports indicate that stuttering involves a transient disruption of the connection between intention and motor action. In this way stuttering is similar to FOG, but also to dystonia and tremor.

#### 1.4.4 Reactions to motor dysfunction

##### 1.4.4.1 Reactions to FOG and dystonia

When the motor system fails to respond as intended, such as during FOG or in dystonia, individuals may attempt to overcome the block. In FOG, patients often push harder to move, but increased effort can exacerbate the freezing (Fahn, 1995). The FOG contractions and the voluntary attempts to overcome them interact dynamically. More specifically, dystonia is often accompanied by *dystonic tremor*, which occurs when patients try to counteract dystonic contractions by moving the affected body part in the opposite direction (Gironell and Kulisevsky, 2009).

##### 1.4.4.2 Reactions to stuttering

As with the reactions to symptoms of FOG or dystonia, people who stutter often react to their stuttering. For example, when the airway is blocked (e.g., by laryngeal closure), the typical response appears to be to push, thereby increasing muscular tension (Van Riper, 1982; Yairi and Seery, 2023).

Accessory movements, often involving the face, head, or trunk, are common in stuttering. According to an influential theory proposed by Brutten and Shoemaker (1971), these movements are learned reactions. Brutten and Shoemaker suggested that these behaviors are learned through operant conditioning, as the maneuvers were assumed to initially help release blocks but later become automatic. In contrast, Yairi and Seery (2023) argued that many of these movements instead may be inherent to stuttering, rather than learned. In an attempt to describe the nature of accessory movements in stuttering, Riva-Posse et al. (2008) analyzed video from 85 people who stutter. All “abnormal” movements were classified as voluntary (i.e., starters or unblockers) or involuntary. Movements classified as involuntary were 2.7 times more frequent than movements classified as voluntary. Most involuntary movements involved the face (e.g., eye closure, jaw movements) or neck, with only one case involving the trunk and none involving arms or hands. Voluntary movements, though less common, often involved the hands (e.g., hand slapping). These findings suggest that involuntary movements in stuttering primarily affect muscles adjacent to speech mechanisms, while movements assumed to be used as starters or unblockers more often involve the hands.

#### 1.4.5 Brain findings and pharmacology

##### 1.4.5.1 Localization of brain lesions

Both dystonia and stuttering can result from brain lesions, particularly within the basal ganglia. For dystonia, the sensorimotor putamen is the most common lesion site (Corp et al., 2022; Stephen et al., 2023). Similarly, Ludlow et al. (1987) found that brain lesions leading to acquired stuttering most often involved the basal ganglia, and Theys et al. (2024) specified this location to the left putamen. Furthermore, a large analysis of gray matter volume in preschool children who stutter (Chow et al., 2023), also highlighted the left putamen as a key structure. These findings align with earlier proposals of the putamen's central role in stuttering (Alm, 2004).

##### 1.4.5.2 Relation to dopamine

The basal ganglia's dependence on dopamine is well-established, particularly in Parkinson's disease, where dopamine depletion in the putamen is central (Gasser and Wichmann, 2023). Dopamine dysregulation is also implicated in dystonia, with low dopamine activity increasing the risk of dystonia (Stephen et al., 2023). Stuttering shows similar links to dopamine, with medications affecting the dopamine system often influencing stuttering, though the responses are very heterogeneous (Alm, 2004). For example, D2 receptor antagonists have shown mixed results, improving stuttering in some cases (Maguire et al., 2000, 2004; Turk et al., 2021) but worsening it in others (Atay et al., 2014). Dopaminergic stimulants also yield inconsistent effects, from stuttering as a side effect (Alpaslan et al., 2015; Ekhart et al., 2021) to reducing it (Devroey et al., 2012; Bodur et al., 2014; Rabaeys et al., 2015; SheikhBahaei et al., 2022).

##### 1.4.5.3 Cortical motor thresholds and intracortical modulation

Considering that people with dystonia and people who stutter often show involuntary movements, one might expect a low motor threshold in these conditions. However, on the contrary, both in dystonia and in stuttering, the cortical motor thresholds tend to be slightly elevated (Mavroudakis et al., 1995; Alm et al., 2013; Chang et al., 2019), as measured using transcranial magnetic stimulation (TMS). In stuttering, this effect has been reported to be specific to the left hemisphere (Alm et al., 2013).

Furthermore, both dystonia and stuttering show impaired modulation of *intracortical inhibition* during motor activity. The inhibition of movements should be reduced before a movement is started. Stuttering individuals have been reported to fail to reduce inhibition in the motor cortex during speech, a pattern also seen in focal hand dystonia during finger tasks (Stinear, 2004; Elfers et al., 2019). In summary, these results suggest insufficient motor cortex preparation in stuttering.

##### 1.4.5.4 Links to impairment of energy metabolism

It has been shown that dysfunctions of the mitochondria can contribute to dystonia (Stephen et al., 2023). The mitochondria are central to the aerobic energy supply. In parallel, it has been proposed that stuttering may be related to limitations in the energy supply to neurons (Chow et al., 2020; Alm, 2021; Boley et al., 2021).

#### 1.4.6 Summary: neurological conditions overlapping with stuttering

The review highlights significant overlap between stuttering and known neurological movement disorders, including task-specificity and situational variability—features often attributed to psychological causes. Stuttering shares notable similarities with motor block symptoms (e.g., freezing of gait in Parkinson's disease) and task-specific dystonia, as well as dystonic tremor. These parallels are supported by overlapping brain findings, particularly involving the basal ganglia and dopamine dysregulation.

A common theme across these conditions is the loss of volitional control and the sensation of being “stuck.” Attempts to overcome this state, such as volitional pushing, can increase tension and sometimes trigger tremor, further linking stuttering to these neurological disorders.

### 1.5 Negative and positive motor signs

In neurology, the symptoms of movement disorders are classified as *negative* or *positive* motor signs. Positive signs refer to excessive or involuntary muscle activity, while negative signs refer to a reduction or absence of the normal muscle activation. If applying this terminology to stuttering, it is clear that stuttering often involves positive motor signs, such as elevated muscular tension and involuntary movements—including blockage of the airflow and tense fixed articulatory postures.

The possibility of negative motor signs in stuttering is less clear, and does not appear to have been explicitly discussed in the literature. Negative motor signs in stuttering would refer to insufficient or absent activation of one or more speech-related muscles necessary for fluent speech. This may be understood as an inability to execute the required motor program (Guenther, 2016, p. 107) for the subsequent phoneme, either partially or completely.

In the following sections, indications of negative and positive motor signs in stuttering will be discussed. This terminology will also be applied in the results section, as a way of structuring the observations.

### 1.6 Negative motor signs in stuttering

#### 1.6.1 Stromsta and Fibiger: part-word repetitions

In the 1970s, Courtney Stromsta and Steen Fibiger investigated the physiological aspects of stuttering using EMG of articulatory muscles. The main hypothesis was that stuttering arises from insufficient anticipatory coarticulation (Stromsta, 1986). In particular, they were interested in part-word repetitions—i.e., when syllables or phonemes are truncated by the disruption. In order to study anticipatory lip coarticulation, they utilized words beginning with consonants that do not require lip involvement, followed by a vowel requiring lip rounding. An example is the word “true.”

Their findings, presented at conferences (Stromsta and Fibiger, 1980; Stromsta, 1987), indicated that part-word repetitions (e.g., /tr e tr e tr e tru:/) tended to lack anticipatory coarticulation during disrupted attempts but show normal coarticulation during successful attempts. Compared to fluent segments, the disrupted segments showed only about 40% of the anticipatory EMG amplitude of the OO lip muscle. Stromsta interpreted this as a failure in planning anticipatory coarticulation, resulting in disruption when the vowel requiring lip rounding should be articulated.

However, the hypothesis that stuttering is linked to impaired planning of coarticulation is not supported by studies of fluent speech in stuttering individuals. During fluent speech, no significant differences in anticipatory coarticulation have been shown between stuttering and non-stuttering individuals (Chang et al., 2002; Sussman et al., 2011). This shows that stuttering individuals do not generally exhibit poor coarticulatory planning, but may experience occasional failures during stuttering events. Therefore, an alternative interpretation is here proposed: The phenomena observed by Stromsta and Fibiger reflects difficulties executing speech motor programs, resulting in weak or absent activation of specific speech muscles. In this case the planning of coarticulation is normal, but the execution of the plan partly fails.

#### 1.6.2 Low or normal levels of speech muscle activation

The proposal that stuttering can be related to insufficient muscular activation aligns with the results from Walsh and Smith (2013). They studied lip muscle activation in children who stuttered, using EMG. It was found that the stuttering children showed 26% lower EMG activity of the lower lip during disfluent speech compared to fluent speech, and 14% lower EMG during fluent speech compared to typically fluent peers.

In adults, Denny and Smith (1992) reported that only 6 of 14 adults showed notably elevated levels of muscular tension during stuttering. In other words, 57% of these adults did not show elevated tension in the muscles that were investigated. It is quite possible that this study underestimated the portion of adults who stutter that have elevated tension during stuttering, but it still supports that high levels of muscular tension is not a universal aspect of stuttering.

In summary, it is here proposed that the studies by Stromsta and Fibiger, and by Walsh and Smith, indicate that speech disruptions in stuttering can be associated with negative motor symptoms, with insufficient activation of certain muscles necessary for speech.

### 1.7 Positive motor signs in stuttering

While the previous section focused on stuttering linked to *insufficient* muscular activity, stuttering is often associated with *excessive* muscular tension and involuntary movements of the speech apparatus—positive motor signs. Elevated tension has long been viewed as central to stuttering. For example, Wendell Johnson defined stuttering as an “*anticipatory, apprehensive, hypertonic* [increased tension]*, avoidance response*” (Johnson et al., 1956, p. 217).

#### 1.7.1 Tremor/oscillations during moments of stuttering

Van Riper (1963, p. 330) reported that swift oscillations in speech muscles often appear when an articulatory posture becomes tense, and he described the oscillations as tremors. Much of the EMG research on stuttering since then has focused on this aspect (e.g., Welsh, 1970; Fibiger, 1971; Smith, 1989; Denny and Smith, 1992). The core tremor frequencies in these studies is about 7–9 Hz, but the range can extend to about 5–15 Hz. Fibiger (1971) reported that stuttering tremor in antagonistic muscles often are synchronous, resulting in co-contraction. Synchronized oscillations often appear in several muscles simultaneously, suggesting that some central process has widespread influence (Denny and Smith, 1992). Smith et al. (1993) showed that such oscillations can also involve laryngeal muscles.

It seems that tremor develops with time in some individuals who stutter, often during school-age years. For example, Kelly et al. (1995) did not find tremor in any of six children age 2.6–7.8 years, but in three of three children age 10–14 years. Similarly, Walsh and Smith (2013) did not detect tremor in any of 64 stuttering children age 3.4–5.9 years. In stuttering adults, Smith (1989) and Denny and Smith (1992) found tremor in a total of 12 of 24 persons, i.e., 50%.

The study by Denny and Smith (1992) showed a strong link between tremor and elevated levels of tension during stuttering. In their study, five of six adults showing elevated muscular tension during stuttering also showed tremor. In contrast, only one of eight adults without notable elevated muscular tension also showed tremor. Interestingly, this last person showed such oscillations both during stuttering and perceptually fluent speech, suggesting a different tremor mechanism in this case.

#### 1.7.2 Laryngeal activity in stuttering events

Studying the role of the larynx in stuttering entails practical difficulties related to its location. Mainly, three methods have been used: (1) *Intramuscular laryngeal EMG*—a highly invasive and challenging method; (2) *endoscopic video*—through the nose; and (3) *electroglottography (EGG)*—non-invasive by registration of the impedance through the larynx.

##### 1.7.2.1 Studies using intramuscular laryngeal EMG and endoscopic video

In the 1970s, Freeman and Ushijima (1978) performed a series of case studies using intramuscular laryngeal EMG and endoscopic filming. Their overall conclusion was that stuttering events in these cases tended to be associated with excessive and/or dysfunctional muscular tension in the larynx. Only in one case, with severe stuttering, the researchers managed to place EMG electrodes both in the opening and the closing muscles. All of these muscles showed high level of activity during moments of stuttering, often with co-contraction of the antagonistic muscles.

*Silent periods*. Freeman and Ushijima observed that speech can be disrupted both by excessive closure, stopping the air flow, and by opening of the larynx, with the vocal folds being too far apart to vibrate. These mechanisms were observed during silent periods before utterances. According to the authors, when the co-contraction was terminated it was almost invariably followed by a fluent sounding utterance. It should here be emphasized that laryngeal disruptions did not only occur as a result of excessive closure, but also as a result of excessive opening. This type of opening disruption does not seem to be generally recognized in textbooks on stuttering, which may be a significant omission. It seems important to introduce terms such as “laryngeal opening” vs. “laryngeal closing” to describe the nature of speech disruptions in stuttering.

*Prolongations and repetitions of sounds*. An observation that may be unexpected was that prolongations of voiceless sounds sometimes appeared to have emerged from dysregulated laryngeal muscles (observed by means of a combination of EMG and endoscopic video, Freeman, 1979). For example, a prolongation of an initial /s/ may be the result of a separation of the vocal folds, hindering the onset of phonation for the following vowel. When the opening muscle relaxed, the vocal folds came into position for phonation and the utterance could continue with the vowel.

In continued case studies using intramuscular laryngeal EMG, by Shapiro (1980) and Shapiro and DeCicco (1982), the basic findings of Freeman et al. were confirmed, with observations of excessive muscular activity during and before stuttering events, and abnormal co-contraction of antagonistic muscles. The excessive tension was often not limited to the larynx, but also involved the tongue and lip muscles. The authors also noted that it was difficult, both for the stuttering person and for listeners, to correctly judge the predominant locus of muscular tension (for example, the larynx vs. the tongue).

Ramig (2004) published an endoscopic video of the larynx and the pharynx of a single case. The video showed that laryngeal blocks in this case were associated with total constriction of the supraglottal structures, with the vestibular folds. The constriction completely blocked the airflow above the vocal folds. Similarly, Bohnen (2011) observed such supraglottal constrictions in four of six adults. These observations indicate that stuttering involving closure of the airflow at the level of the larynx frequently involves supraglottal closure and not primarily hard closure of the vocal folds. It can be added that in one person, Bohnen observed difficulties initiating phonation because of separated vocal folds.

##### 1.7.2.2 Electroglottography (EGG)

EGG has the advantage of being non-invasive, but the disadvantage of providing more limited information. Conture et al. (1986) studied children who stuttered, age 3.1–5.9 years, and found indications of difficulties initiating and sustaining phonation. In addition, it was observed that some young children who stutter have difficulties with laryngeal muscular control also when speech was perceived as fluent by the listeners.

### 1.8 Studies presented in this article

Building on the aspects discussed in Sections 1.1–1.3, three initial studies were conducted as undergraduate theses at Uppsala University under the supervision of Per Alm. The overall aims of the studies were to:

1. Develop non-invasive, multimodal recording methods for speech in people who stutter.
2. Create effective methodologies for presenting and analyzing multimodal speech data.
3. Explore the immediate mechanisms of speech disruptions during stuttering events, through qualitative analysis of physiological measures, as a basis for subsequent quantitative studies of larger cohorts.

## 2 Method

### 2.1 Description of studies

The three initial studies, as undergraduate theses, were conducted in 2015 and 2019, here labeled as Studies A (Johansson and White, 2015), B (Brösemyr et al., 2019), and C (Grinde and Olsson, 2019). Study A included surface EMG of muscles closing and opening the lips, EGG, and formant analysis, with a particular focus to replicate the results by Stromsta and Fibiger (1980) and to explore tension and tremor during stuttering events. Study B focused on exploring the possibilities of using surface EMG on the tongue. In Study C, endoscopic video of the vocal folds was combined with EGG. In study A and B, video of the face was included.

The studies focused on production of overt speech, elicited by reading aloud (words, sentences, continuous texts), description of pictures, and spontaneous speech on various topics. Some of the words to read were chosen because the first vowel required lip rounding, preceded by consonants not requiring lip articulation. The purpose of these words was to facilitate the analysis of anticipatory coarticulation, according to Stromsta and Fibiger (1980).

It should be emphasized that the analysis of the data has been qualitative, focusing on detailed analysis of single stuttering events rather than quantitative statistical approaches. The analyses in the undergraduate theses served as a first step, and was followed by more detailed and comprehensive exploratory analysis. The presented figures have been created and interpreted for this article, based on the data and analyses in the undergraduate theses. Future larger-scale studies are needed to investigate the prevalence of various motor signs, the existence of subgroups or clusters, etc.

The presented results consist of examples of stuttering events, showing various speech motor anomalies. The purpose is, as far as possible, to understand the dynamics of moments of stuttering. In particular, the analysis has aimed to characterize the first observable motor anomalies in a stuttering event—the event that may result in a reaction.

### 2.2 Participants

All participants self-identified as stuttering, and their symptoms were verified by the experimenters. The exclusion criteria were: Other known neurological disorders, reading difficulties, known hearing impairments, and < 1% stuttered syllables (SS). The study protocols were approved by the institutional review board and the Regional ethics committee (2010/208, 2018/221). Written informed consent was obtained from all participants in accordance with the Declaration of Helsinki.

In Study A, 14 adults were initially included, but 3 were excluded because of < 1% SS (i.e., 21% exclusion rate). The age of the remaining 11 participants ranged from 19 to 57 years (mean 35.4, 2 females and 9 males). The Stuttering Severity Index-4 score (SSI-4, Riley, 2008) ranged 13.5–34 (mean 24), see Table 1. The participants are labeled A1, A2, etc.

**Table 1:** Participants included in Study A, with SSI-4 scores.

**ID**	**Gender**	**Stuttered syllables (%)**	**Physical concomitants**	**Duration**	**Total SSI score**	**SSI label**	**Ratio Phys.Con./%SS**
A2	Male	6.0	0.0	1.5	17.5	Mild	0.00
A4	Male	5.0	1.0	1.2	18.0	Mild	0.20
A5	Male	13	1.0	1.5	25.5	Moderate	0.08
A6	Female	8.7	1.5	1.1	21.0	Mild	0.17
A7	Male	7.6	3.0	5.1	29.0	Moderate	0.40
A8	Male	17	5.0	8.4	32.5	Severe	0.30
A9	Male	28	2.0	3.0	29.0	Moderate	0.07
A10	Female	3.3	4.0	2.0	19.0	Mild	1.21
A12	Male	1.7	1.0	0.9	13.5	Very mild	0.59
A13	Male	2.5	1.0	1.4	14.0	Very mild	0.41
A14	Male	26	5.0	5.1	34.0	Severe	0.19

“Duration” is the mean duration of the three longest stuttering events. SSI physical concomitants are rated from 0 to 5. The ratio in the right column is the Physical Concomitants score divided by the percentage stuttered syllables.

Study B and C included a total of 8 adults (not included in study A), labeled BC1, BC2, etc. The age range was 24–53 years (mean 31.5, 4 females and 4 males). Study B included 7 of these participants, and study C included 6. Examples from three participants are included in this article: BC6 (female), BC7 (female), and BC11 (male).

### 2.3 Recording of signals related to stuttering events

Ideally, comprehensive data on muscular activity and movements from all levels of the speech process should be available for analysis—breathing, laryngeal behavior, tongue, soft palate, jaw, and lips. However, due to practical limitations and concerns for participant wellbeing, each recording was restricted to a limited set of modalities. The following measures and indicators were used in these studies:

- *Surface EMG of the orbicularis oris* (OO): Indicating lip rounding and mouth closure.
- *Surface EMG of the depressor labii inferioris* (DLI): Indicating mouth opening through the lowering of the lower lip.
- *EMG of the tongue's dorsal surface*: Indicating tongue muscle activity.
- *EGG*: Indicating characteristics of phonation.
- *Endoscopic video of the larynx*: Visualizing glottal opening and closure as well as pharyngeal constriction (not stroboscopic video).
- *Broadband sound spectrograms*: Showing formant movements, with F2 particularly indicating tongue movement or fixed positioning.
- *Video of the face*: Capturing jaw and lip movements, as well as facial tension indicators.
- *Sound recordings*: Providing perceptual indicators of the speech processes.

Surface EMG was recorded using Biopac MP100 (BIOPAC Systems, Inc.), with 1,000 samples per second and hardware filters for 10 Hz high pass, 500 Hz low pass, and 50 Hz notch. In Study A, EasyCap E271 circular flat 4-mm Ag/AgCl electrodes were used, following guidelines from Lapatki et al. (2003), with a 10-mm center distance, applied using double-sided tape (5000NS, Nitto, Inc.) with punched holes, Fixomull Stretch (BSN Medical, Inc.), skin preparation gel (NuPrep, Weaver, Inc.), and EC2 adhesive electrode paste (Grass, Inc.). In Study B, self-adhesive disposable electrodes (Neuroline 70015-K/12) were used, including applications to the tongue surface.^2^ The adhesive surface of the electrodes was trimmed to appropriate sizes. Impedance was kept below 10 kΩ for all EMG measurements. EGG signals were recorded using the Glottal Enterprises EG2-PCX system, paired with sound recordings via the AVID M-Audio Fast Track C600 external sound card. Facial video recordings were captured at 25 frames per second with a resolution of 720 × 576 pixels. Endoscopic video of the vocal folds was recorded using the Xion OR-PC 211 video endoscope.

### 2.4 Signal processing and qualitative data analysis

#### 2.4.1 Post-processing

The EMG signals were high-pass filtered (20 Hz) and rectified using AcqKnowledge 4.4 software (BIOPAC Systems, Inc.). The left and the right muscle waveforms were merged to the mean rectified waveforms for the OO and the DLI muscles, respectively, and smoothed using 5 ms moving average. All physiological signals were scaled before being exported as a multichannel WAV file. The WAV file containing the EMG recordings was merged with the WAV file containing the sound and EGG recordings, using the audio editor Sony Sound Forge Pro 10.0. This merged WAV file was synchronized with the video file by aligning the start time of the WAV file with that of the video, based on a clapperboard index in the sound waveform. Sony Sound Forge Pro 10.0 enables simultaneous video and audio playback while displaying the waveforms of multiple channels. By opening the video file first and importing the waveforms from the synchronized multichannel WAV file, it was possible to view EMG and EGG waveforms alongside audio and video. The software also allows marking and saving regions of interest with annotations for coding and further analysis. Additionally, for some of the data, broadband spectrograms were generated using Wavesurfer 1.8.8.p4 (http://www.speech.kth.se/wavesurfer/), with Blackman window and 220 Hz bandwith.

#### 2.4.2 Analysis of spectral power of tremor/oscillations

For each individual in the stuttering group in Study A, 20 segments of stuttered speech were extracted from the recordings and down-sampled to 1 kHz. The selection of segments was not random, because the purpose was to find examples of tremor/oscillations for qualitative analysis. Priority was given to segments showing indications of tremor or high levels of muscular tension.

For analysis of the peak frequency of oscillations, the frequency of the rectified EMG peak spectral power between 7 and 15 Hz was calculated for each segment. The lower cut-off, 7 Hz, was selected to avoid interference from normal speech movements. Spectrogram power was calculated using the Peak Matched Multiple Windows (PMMW; Hansson and Salomonsson, 1997; Hansson, 1999) with a sequence length of 512 ms, time-shift of 16 ms, and FFT-length of 8,192 samples. The number of multitapers is 4, corresponding to a frequency resolution of ~7 Hz.

## 3 Results

The results section is divided based on negative and positive motor signs, as a way to structure the observations. The selection of examples was made in order to provide an overview of various types of disruptions and various locations within the vocal tract. The first part, on negative motor signs, is focused on part-word repetitions and the methodology of Stromsta and Fibiger (1980). The reason for this choice is that negative motor signs are, by definition, difficult to show—if there is no muscular activity, it might be because the speaker is pausing. The type of disruptions (repetitions) reported by Stromsta and Fibiger are particular, in that the muscular activity is missing only in some of the required speech muscles, and that it can be shown that fluency is restored when this muscle is activated. Therefore, for methodological reasons, the section on negative motor signs is limited to this type of disruption. The second part, on positive motor signs, shows examples of sustained tension, tremor, supraglottal contraction, etc. The individual examples will be discussed within the results section, to be followed by an overall discussion in the subsequent section.

### 3.1 Negative motor signs: analysis of part-word repetitions (Study A)

#### 3.1.1 Participants and severity profiles

Based on the report by Stromsta and Fibiger (1980), an inclusion criterion for this analysis was that the stuttering should include repetitions of incomplete syllables. Among the 11 adults in Study A, five were judged to exhibit frequent part-syllable repetition and were therefore included in the continued analysis of this aspect. Out of these five, at least three were considered to show the negative motor sign described by Stromsta and Fibiger (1980): Missing or weak anticipatory muscular activation during disrupted attempts, with normal anticipatory activation in the successful attempt. However, we later found that this type of negative motor sign can also occur in repetitions of complete syllables, see Figures 2, 3.

The mean SSI-4 severity score for the three persons showing negative signs was 24, which was similar to the other participants, with a mean score of 23. However, the profiles of the SSI subscores differed between these two groups. Those with negative motor signs showed relatively few physical concomitants, with a mean rating of 1.0 to be compared to 2.9 for the others (rating from 0 to 5). In contrast, the mean percentage of stuttered syllables was higher for the group showing negative signs, with 15.6% compared to 10.2% for the rest. When calculating a ratio between the physical concomitants score and the percentage of stuttered syllables (see Table 1), all three with the negative motor signs had ratios below 0.08, while the others ranged from 0.17 to 1.2.

#### 3.1.2 Participant A5 “kråkans”: truncated syllable and missing articulation

*Figure*: 1.

*Participant*: A5, male (SS: 13%, PhysScore: 1, moderate).

**Figure 1:**
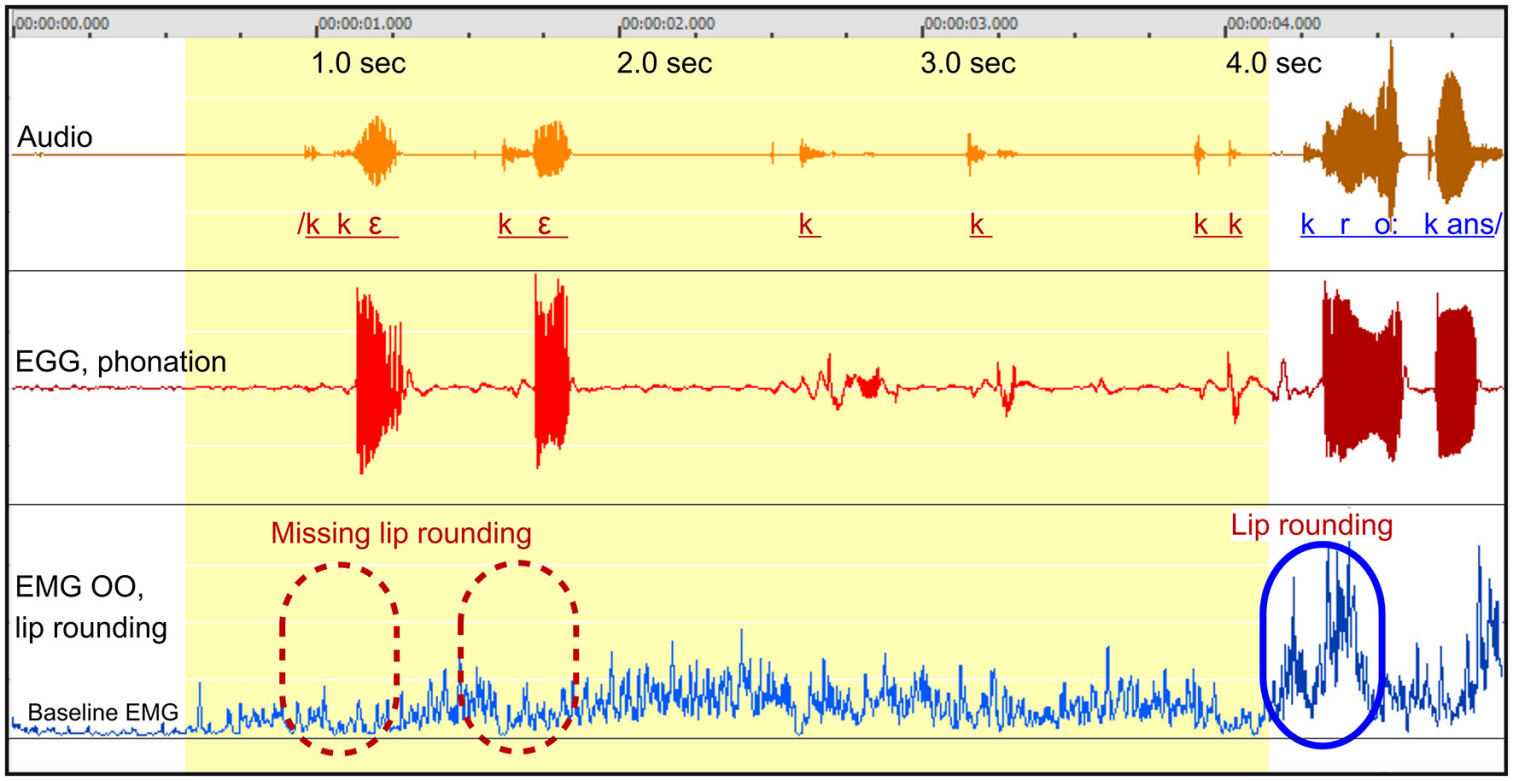
See Section 3.1.2. Male participant A5 attempting to say the Swedish word *kråkans*. The yellow segment indicates the duration of the stuttering event. Transcription in red represents the stuttered part, while the transcription in blue designates the perceptually fluent utterance. The blue marker (solid line) in the lower right indicates the anticipatory coarticulation prior to the fluent vowel. The dashed red markers represent the expected location of the missing anticipatory lip rounding. Arbitrary units.

*Stuttering event*: Figure 1 depicts an attempt to say the Swedish word *kråkan*. The yellow segment represents the stuttered portion, with approximately five repeated attempts, followed by the successful attempt shown to the right. The interval between repetitions was about 0.6 s.

*Missing anticipatory lip rounding*: The blue marker to the lower right shows the contraction of the OO muscle needed for the /o/ sound. In contrast, the dashed red markers show the location of the expected OO activity in the interrupted attempts, which is missing.

*Normal phonation*: The EGG waveform shows normal phonation, which is interrupted about 0.3 s after the expected time for the missing OO activity.

*Elevated sustained OO activity*: The OO waveform to the left shows the baseline resting level. It can be noted that the OO activity is moderately elevated during the entire stuttering event but without any notable peaks related to the speech attempts. The sustained elevated activity decreases just before the end of the stuttering event, followed by proper muscular activation.

*Tongue articulation*: Formant frequency analysis of the first two attempts revealed incorrect positioning of the tongue, with a second formant (F2) frequency of ~1,900 Hz instead of the expected 860 Hz.

*Summary*: This event displays negative motor signs in the form of missing anticipatory lip rounding, and a positive sign in the form of sustained unspecific OO activity. The position of the tongue was incorrect while phonation was normal. This constellation of symptoms is consistent with a “motor block”: The OO muscle is temporarily unresponsive, resulting in a combination of missing speech-specific activation and the addition of unspecific dysfunctional activation. Notably, this person also showed stuttering events dominated by positive motor signs, see Figure 10.

#### 3.1.3 Participant A2 “skuret”: missing articulation after vowel

*Figure*: 2.

*Participant*: A2, male (SS: 6%, PhysScore: 0, mild).

**Figure 2:**
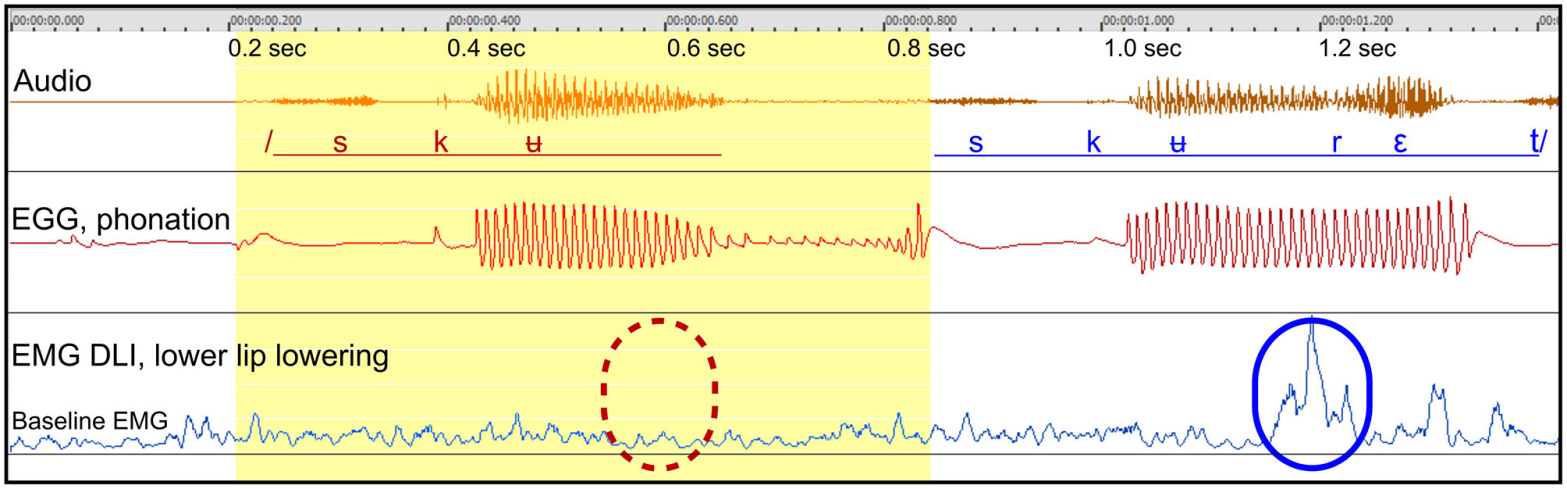
See Section 3.1.3. Male participant A2 producing the Swedish word *skuret*, with one part-word repetition. The blue marker (solid line) in the lower right shows the activity of the DLI in a fluent utterance, for the opening of the mouth after the /ʉ/ sound. The dashed red marker indicates the expected point of missing DLI activation in the disrupted attempt.

*Stuttering event*: Figure 2 shows an attempt to produce the Swedish word *skuret*. A part-word repetition is observed, highlighted by the yellow segment.

*Missing DLI activation after rounded vowel*: The blue marker (lower right) shows the activity of the DLI muscle in the final fluent utterance, for mouth opening following the /ʉ/ sound. The dashed red marker shows the expected location for DLI activation during the interrupted attempt.

*Normal phonation*: When comparing the EGG waveforms between the disrupted and the successful attempts, the waveforms are basically identical up to 0.13 s after the onset of phonation, when the activation of the DLI is expected. From this point in time, the amplitude of the phonation begins to fade and the word is restarted.

*Summary*: The failure to activate the DLI muscle is the only anomaly observed in the disrupted attempt, and the disruption begins at the moment when the activation of the DLI should have occurred. The F2 formant frequency was roughly correct, indicating correct positioning of the tongue. Based on the available data, it appears that speech was disrupted and restarted in order to repair the missing articulatory activity.

#### 3.1.4 Participant A9 “skolan”: missing articulation after vowel

*Figure*: 3.

*Participant*: A9, male (SS: 28%, PhysScore: 1, moderate).

**Figure 3:**
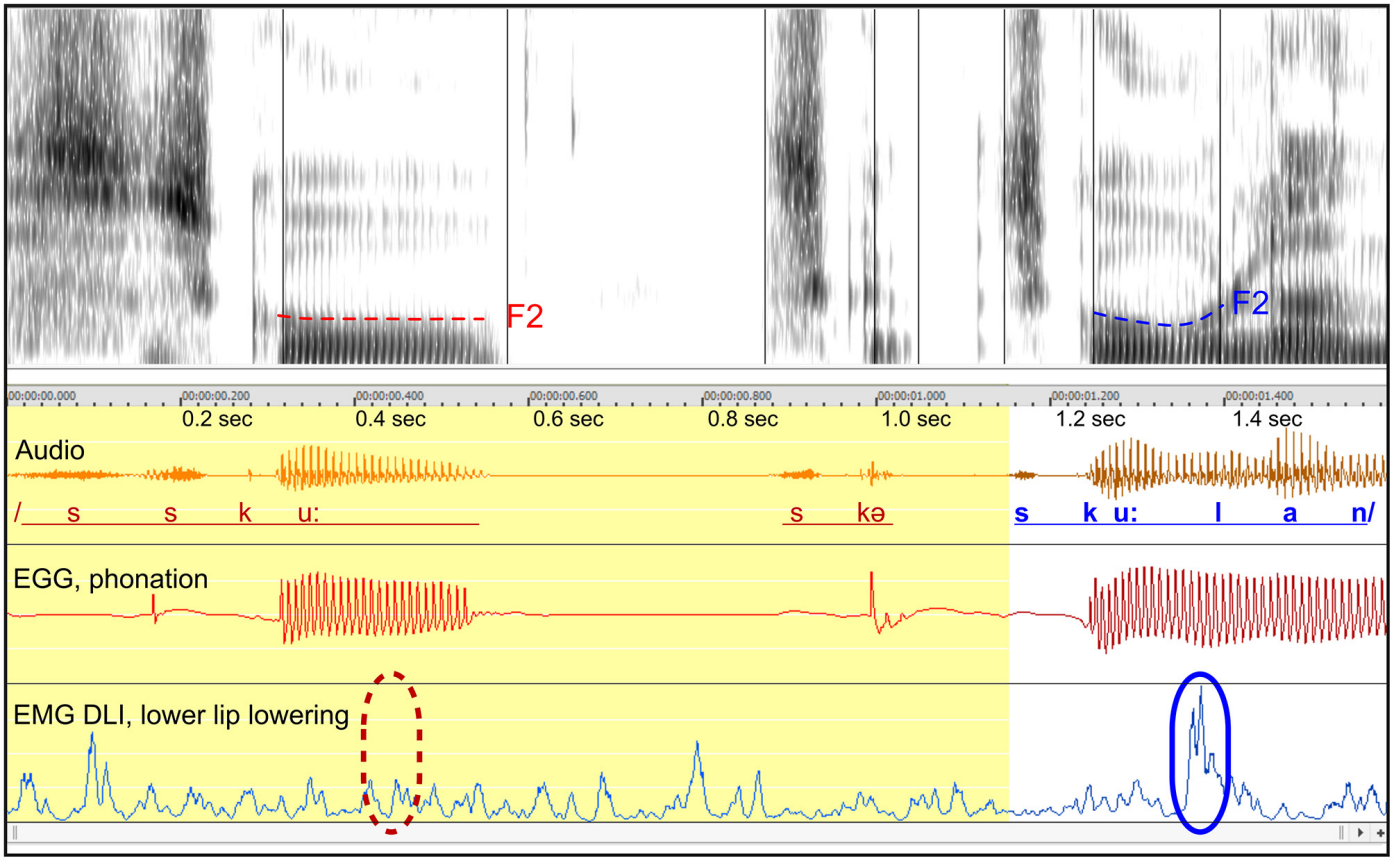
See Section 3.1.4. Male participant A9 producing the Swedish word *skolan* with two part-word repetitions. The blue marker (solid line) in the lower right indicates the activity of the DLI muscle in the fluent utterance, contributing to the opening of the mouth after the /u/ sound. The dashed red marker in the lower left shows the expected point in time of missing DLI activation in the disrupted attempt. Dashed lines in the spectrogram represent the trajectories of the second formant (F2), likely to indicate tongue movements.

*Stuttering event*: Figure 3 shows an attempt to produce the Swedish word *skolan* within a sentence. Two part-word attempts are followed by a successful attempt.

*Missing DLI activation after rounded vowel*: The blue marker to the right shows the expected DLI activation for mouth opening after the rounded vowel. The dashed red marker shows the expected location in the disrupted attempt.

*Normal phonation*: The phonation in the interrupted attempt is normal, based on the EGG waveform, but is disrupted about 0.12 s after the expected onset of the missing DLI activation.

*Missing tongue movements*: In the spectrogram, the F2 formant is highlighted. In the fluent attempt, the F2 curve bends upwards in preparation for the next sound, reflecting tongue movement. In contrast, the F2 trajectory in the disrupted attempt is flat, indicating that the tongue remains static. The missing tongue movement occurs simultaneously with the missing activation of the DLI.

*Summary*: Similarly to the example in Figure 1, the data indicate that both a lip muscle and the tongue show simultaneous missing muscular activation, while the phonation appears to be normal and is disrupted subsequently. Similarly to Figures 1, 2, this example can be interpreted in terms of a motor block resulting in a negative motor sign, affecting the DLI and the tongue.

#### 3.1.5 Summary: stuttering with negative motor signs in part-word repetitions

In summary, the results from Stromsta and Fibiger's (1980) and our findings, exemplified above, support the proposal that disruptions in stuttering can be associated with missing muscular activation, i.e., a negative motor sign. For methodological reasons, these observations are yet limited to part-word repetitions, but other types of disruptions should also be investigated.

An important observation is that participant A5 (see Figures 1, 10) show a combination of negative and positive signs, also during the same stuttering event (Figure 1). In Figure 1, the positive sign consists of a sustained moderate level of activation, which is largely unaffected by attempts to restart the word. This suggests that the motor circuit controlling this muscle is temporarily unresponsive. In Figure 10, this participant shows tremor of the OO lip muscle—a positive sign. Overall, these symptoms are consistent with the concept of a transient “motor block,” as defined by Giladi et al. (1992). The tremor in Figure 10 might be triggered by volitional efforts to overcome the block, as described by Gironell and Kulisevsky (2009) in dystonia.

### 3.2 Positive motor signs in stuttering: involuntary contractions

In this section, examples of positive motor signs during stuttering events are shown from Studies A, B, and C. The examples are dominated by tremor, but also show sustained contractions of various types, related to fixed postures. It is shown that the overt characteristics of tremor can be very varying, from silent trembling of muscles during fixed postures to audible sound repetitions produced by the tremor.

#### 3.2.1 Study A: lip EMG and EGG

##### 3.2.1.1 Tremor index, Study A

In order to estimate the tendency to display tremor in Study A, a tremor index was calculated based on EMG recordings of the OO muscle, during stuttering events. It was calculated as the ratio between the spectral power of the typical tremor frequencies (7–20 Hz) and the spectral power of the normal articulatory movements (1–5 Hz).

**Figure 4:**
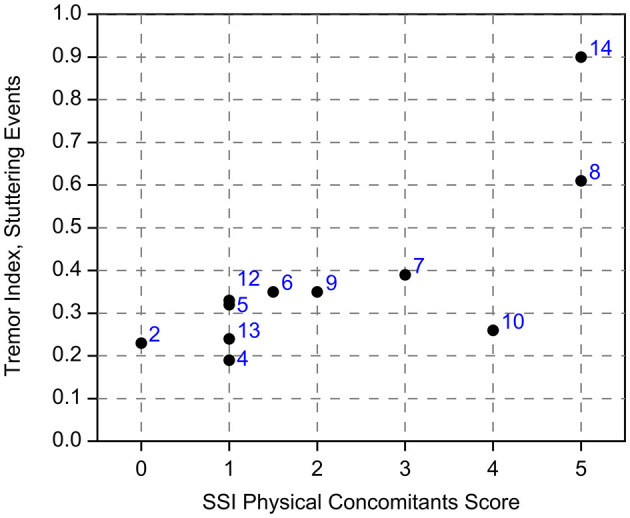
See Section 3.2.1.1. Tremor index for stuttering events, in relation to the SSI Physical Concomitants scores. The numbers indicate the identity of the participants from Study A.

A control group of 13 typically fluent persons showed a tremor index in the range of 0.10–0.29 (median 0.14, mean 0.16). For perceptually fluent speech, the stuttering group had a range of 0.09–0.43 (median 0.14, mean 0.19), and for stuttering events a range of 0.19–0.90 (median 0.33, mean 0.38). The tremor index for stuttering events is correlated with the SSI Physical Concomitants score (*r* = 0.77, *p* = 0.005, Pearson), see Figure 4. This indicates that the most severe physical concomitants are associated with tremor in the 7–20 Hz range. The average peak frequency of tremor was 8.3 Hz, both for the OO and the DLI muscles.

##### 3.2.1.2 Participant A14 “manna”: audible fixed posture, shifting to tremor

*Figure*: 5.

*Participant*: A14, male (SS: 26%, PhysScore: 5, severe).

**Figure 5:**
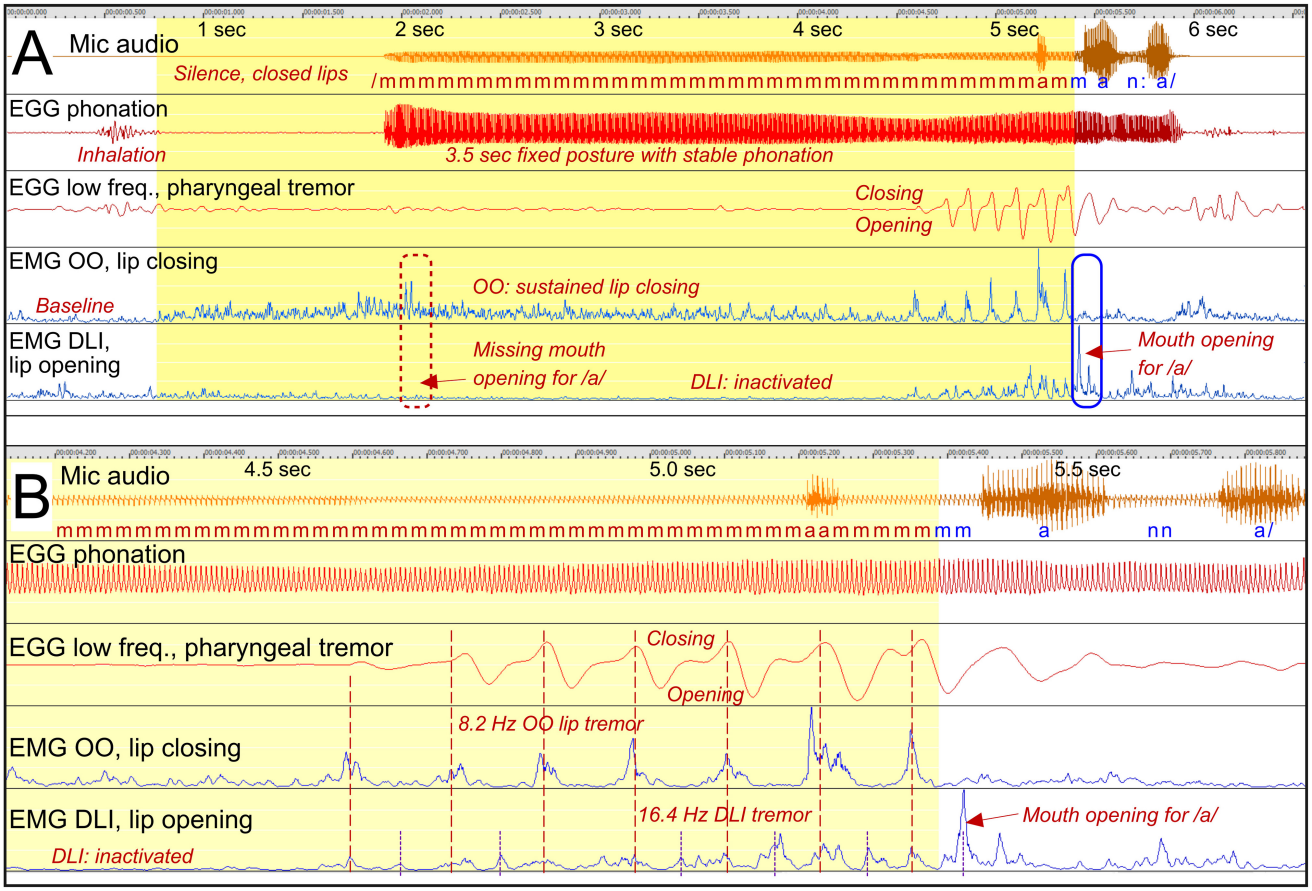
See Section 3.2.1.2. Participant A14. **(A)** Shows a 3.5 s prolongation of /m/ with sustained OO activity, closing the lips and hindering the continuation to the vowel. DLI is inactivated. The blue marker shows the balance between OO and DLI for appropriate mouth opening. Tremor emerges before the resolution of the stuttering event. **(B)** Shows the tremor in more detail. The EGG waveform is shown for two frequency ranges: High-pass filtered showing phonation, and low-pass filtered showing tremor.

*Stuttering event*: Figure 5A, shows a 3.5 s prolongation of /m/, to utter the Swedish word /manna/, with emerging lip tremor at the end. Figure 5B shows the tremor in more detail.

*Sustained OO activity*: The OO EMG waveform shows sustained OO activity (closing lips) beginning about a second before the phonation starts, and is continuous until the onset of the tremor. During this time the DLI (opening lips) is inactive. The activation of the OO and the inactivation of the DLI explains the sustained closure of the mouth and thereby the prolongation of the /m/. The subsequent vowel cannot be produced until the balance between the OO and the DLI is reversed and the mouth opens.

*Emerging tremor*: Figure 5B shows the OO tremor in more detail. The tremor consists of 8.2 Hz oscillations with antagonistic co-contraction of the DLI. However, the DLI also shows a harmonic, at 16.4 Hz. This means that the DLI peaks when the OO is relaxed. At about 5.4 s, this DLI peak is strong enough to result in opening of the mouth, making it possible to proceed with /a/.

*EGG tremor*: It can be noted that also the EGG signal shows 8.2 Hz tremor, though without affecting the phonation. It is therefore unlikely that these slow oscillations in the EGG waveform originate from vocal fold movements, but may instead be the result of pharyngeal contractions or tongue movements.

*Phonation*: The phonation is stable during the whole event.

*Summary*: First, this is an example of stuttering as a result of involuntary sustained contraction of an articulator, resulting in a fixed posture. It appears that the lip muscles are unresponsive during this period. Second, the fixed posture is finally resolved by the emergence of a tremor in the antagonistic lip muscles, with the double frequency in the opening muscle. A peak from the DLI tremor results in mouth opening and continued speech. In this case, the tremor appears to have the paradoxical effect of breaking a fixed posture and facilitating speech. It is possible that the tremor emerges as a result of voluntary efforts to break the fixed posture, similarly to the emergence of dystonic tremor.

##### 3.2.1.3 Participant A14 “bagge”: silent fixed posture with lip closing and tremor

*Figure*: 6.

*Participant*: A14, male (SS: 26%, PhysScore: 5, severe).

**Figure 6:**
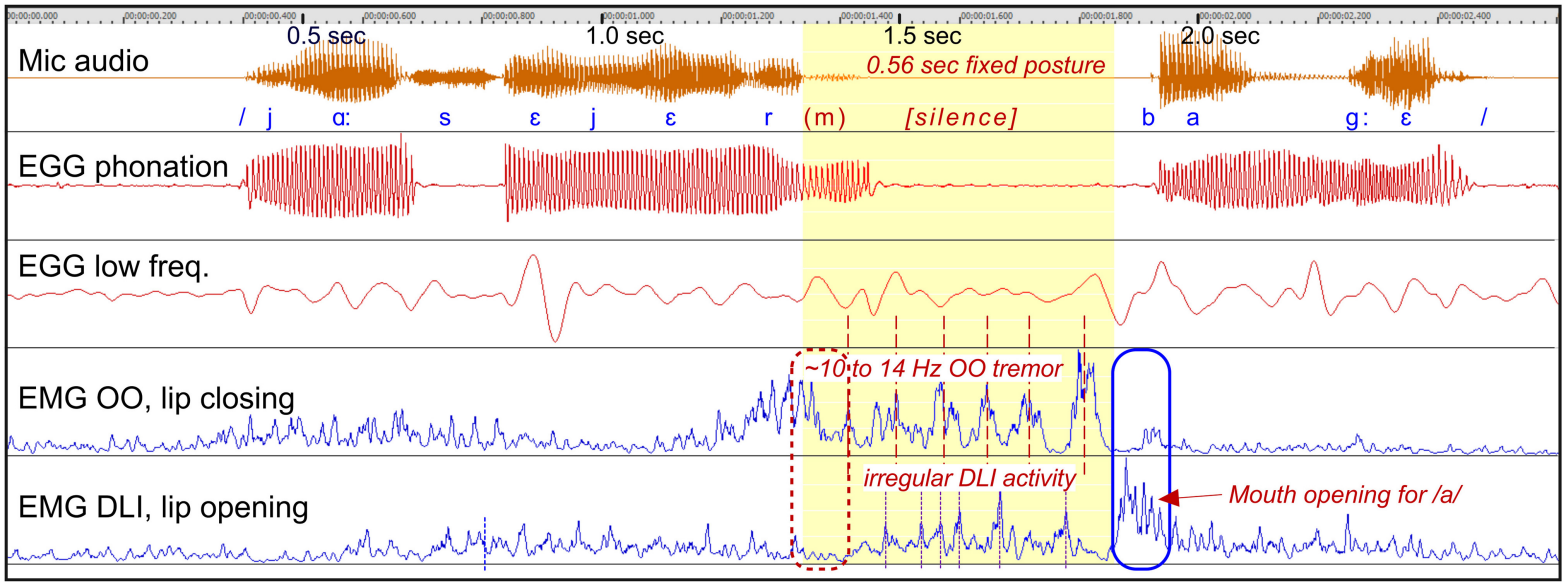
See Section 3.2.1.3. Participant A14. The graph shows a stuttering event characterized by a silent period before the production of /b/, with involuntary prolonged closure of the lips. The OO muscle shows tremor in the range of 10–14 Hz. The stuttering event ends when the tremor ends and the DLI muscle produces an opening contraction, at the blue marker.

*Stuttering event*: Figure 6 shows a 0.56 s silent fixed posture with closed lips, before /b/ in “bagge.”

*Dysfunctional OO and DLI activity*: It appears that the lips get “stuck” in a closed position, with the OO muscle showing 10–14 Hz tremor closing the lips. The expected opening activity of the DLI muscle at 1.3 s is missing, suggesting a combination of positive and negative motor signs. The lips are closed until this tremor stops and strong DLI activity opens the lips: the /b/ is produced and the word continues.

*Summary*: This is an example of the lips getting “stuck,” and both the opening and the closing muscles appears to be in unresponsive states, with tremor.

##### 3.2.1.4 Participant A8 “majskolv”: prolongation with fixed posture, silent tremor

*Figure*: 7.

*Participant*: A8, male (SS: 17%, PhysScore: 5, severe).

**Figure 7:**
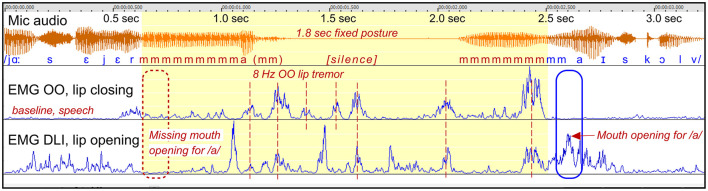
See Section 3.2.1.4. Participant A8. The graph shows a stuttering event with closed lips, characterized by a combination of prolongation of /m/, with sustained OO contraction, and a silent period with 8 Hz lip tremor. The blue marker shows the balance between OO and DLI for appropriate mouth opening.

*Stuttering event*: Figure 7 shows a 1.8 s fixed posture during the articulation of the initial /m/ in “majskolv.” The fixed posture begins and ends with a prolongation of /m/, interrupted by a 1 s silent period with lip tremor.

*Dysfunctional OO and DLI activity*: This example is similar to the example in Figure 6, by another person. Again, it appears that the lips are “stuck” in closed position. The initial sustained OO activity (prolongation of /m/) change into a highly regular 8 Hz OO tremor during the silent period. The DLI show irregular tremor, often with co-contraction in relation to the OO muscle. The stuttering event ends when the OO relax and the DLI can be properly activated for mouth opening.

*Summary*: Similarly to the example in Figure 6 by another person, this is an example of the lips getting “stuck,” and both the opening and the closing muscles appears to be in unresponsive states, with tremor. The event may be described as a “motor block.”

##### 3.2.1.5 Participant A8 “jag”: silent fixed posture with tremor and co-contraction

*Figure*: 8.

*Participant*: A8, male (SS: 17%, PhysScore: 5, severe).

**Figure 8:**
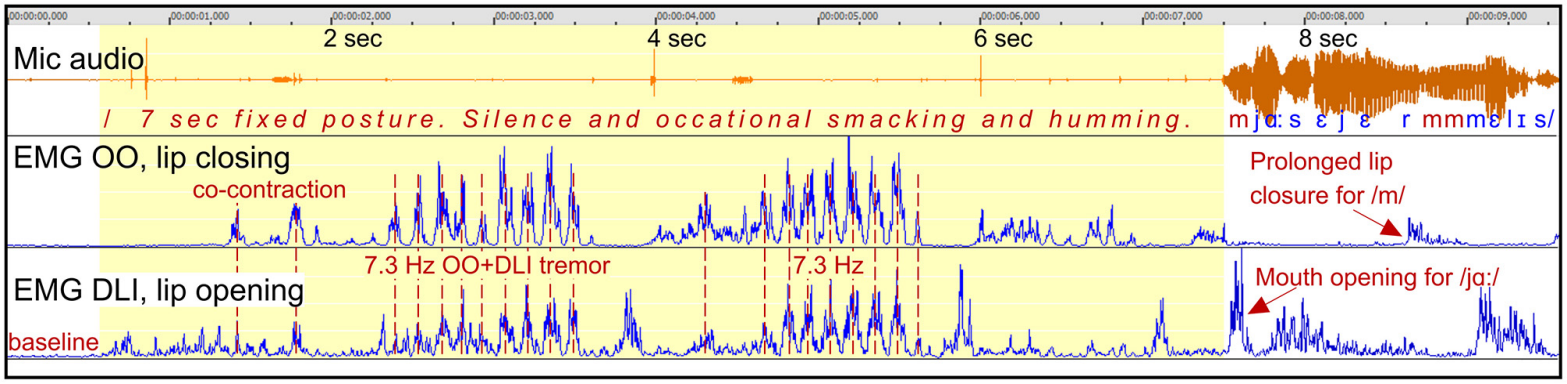
See Section 3.2.1.5. Participant A8. The graph shows a 7 s silent period and fixed posture with closed lips when trying to initiate a phrase beginning with the Swedish word /ja:/. The silent period is characterized by highly regular and strong tremor at 7.3 Hz, with synchronized co-contraction of the closing and opening lip muscles.

*Stuttering event*: Figure 8 shows a 7 s stuttering event before the Swedish word “jag” (/ja:/), constituted by lip closure and silence, except from occasional smacking and humming. The lip closure is dysfunctional as the initial sound /j/ requires open mouth.

*Dysfunctional OO and DLI activity*: The fixed posture with closed lips is primarily maintained by lip tremor at 7.3 Hz, with synchronized co-contraction of the antagonistic OO and DLI muscles. The lips remain closed because of the greater strength of the OO muscle compared to the DLI. The tremor is strong and highly regular. In the video recording, this is visually observable as a trembling in the lips while they are pressed together. The phrase is uttered when the tremor subsided, the OO muscle relaxes, and the DLI is activated in order to open the mouth.

*Summary*: Again, this is an example of fixed posture with the lips “stuck” in closed position. It resulted in a silent period, with no prolongation of sounds. The silent period was dominated by highly regular 7.3 Hz tremor with co-contraction.

##### 3.2.1.6 Participant A8 “barkspån”: audible and silent tremor

*Figure*: 9.

*Participant*: A8, male (SS: 17%, PhysScore: 5, severe).

**Figure 9:**
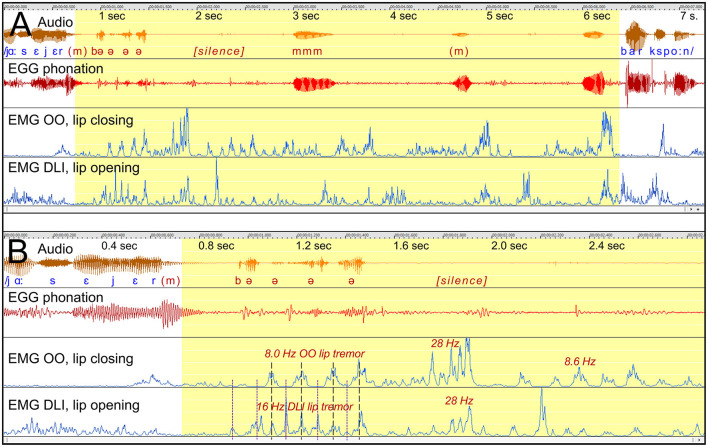
See Section 3.2.1.6. Participant A8. **(A)** Shows a 5.5 s stuttering event before /b/ in the Swedish word “barkspån.” The stuttering event is characterized by lip tremor with varying frequencies and dynamics. **(B)** Shows the tremor during the first half in more detail. The first tremor period consists of 8 Hz OO tremor and synchronized 16 Hz DLI tremor, resulting in audible opening and closing of the lips at 8 Hz. The later tremor shows co-contraction of the OO and the DLI, resulting in silent sustained lip closure.

*Stuttering event*: Figure 9A shows a 5.5 s stuttering event before the Swedish word “barkspån,” with strong lip tremor beginning about 0.4 s after the onset of the event. Figure 9B shows the tremor in the initial part in more detail.

*Dysfunctional OO and DLI activity*: The tremor of the OO and the DLI muscles vary in frequencies and type of interaction. Figure 9B shows that the initial OO tremor is at 8.0 Hz, interacting with a synchronized DLI tremor of the double frequency, 16 Hz. Because the DLI tremor has the double frequency, it has peaks when the OO muscle is inactive. This results in an opening and closing movement of the lips at 8 Hz, with the audible consequence of a repetition of / e /. This is followed by synchronized tremor with co-contraction of the OO and the DLI, first at 28 Hz followed by 8.6 Hz. Because the OO is stronger than the DLI, co-contraction will result in sustained lip closure.

*Summary*: This example is of particular interest because it shows how different tremor frequencies in antagonistic muscles can result in opening and closing of the mouth, with audible stuttering as a result.

##### 3.2.1.7 Participant A5 “blidka”: part-syllable repetition linked to tremor

*Figure*: 10.

*Participant*: A5, male (SS: 13%, PhysScore: 1, moderate).

**Figure 10:**
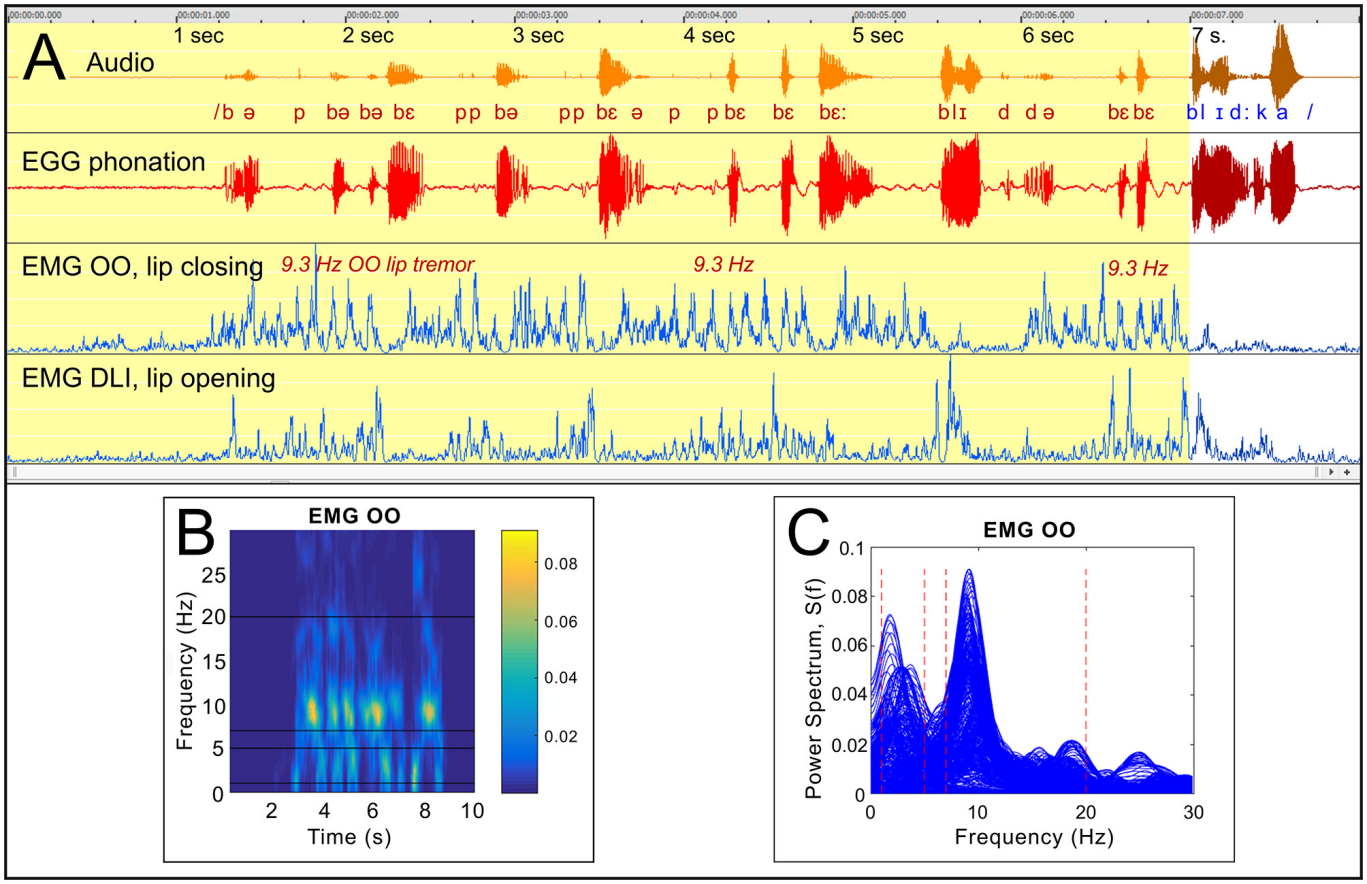
See Section 3.2.1.7. Participant A5. **(A)** Shows a stuttering event with fast part-syllable repetitions before the Swedish word “blidka,” with strong OO tremor at 9.3 Hz. **(B)** shows a time-frequency graph, and **(C)** is a plot of all power spectra for 512 ms sequences of the OO EMG, time-shifted in steps by 16 ms.

*Stuttering event*: Figure 10A shows a 7 s stuttering event with fast part-syllable repetitions of the first sounds in the Swedish word “blidka.” Figures 10B, C show the frequency of the OO muscular activity.

*Dysfunctional OO and DLI activity*: The OO muscle shows a more or less continuous 9.3 Hz tremor during the stuttering event, accompanied by more irregular DLI activity. The word can be produced when the tremor has ended.

*Summary*: The nature of the stuttering in this case differs from the previous examples, with extended fast repetition of syllable fragments. The repeated segments typically include /b/ or /p/, suggesting that the audible stuttering is directly related to the OO tremor, with occasional audible burst when the OO muscle is inactive.

#### 3.2.2 Study B: tongue EMG

##### 3.2.2.1 Participants BC7 and BC11: audible fixed posture with sustained tongue muscle activity

*Figure*: 11.

**Figure 11:**
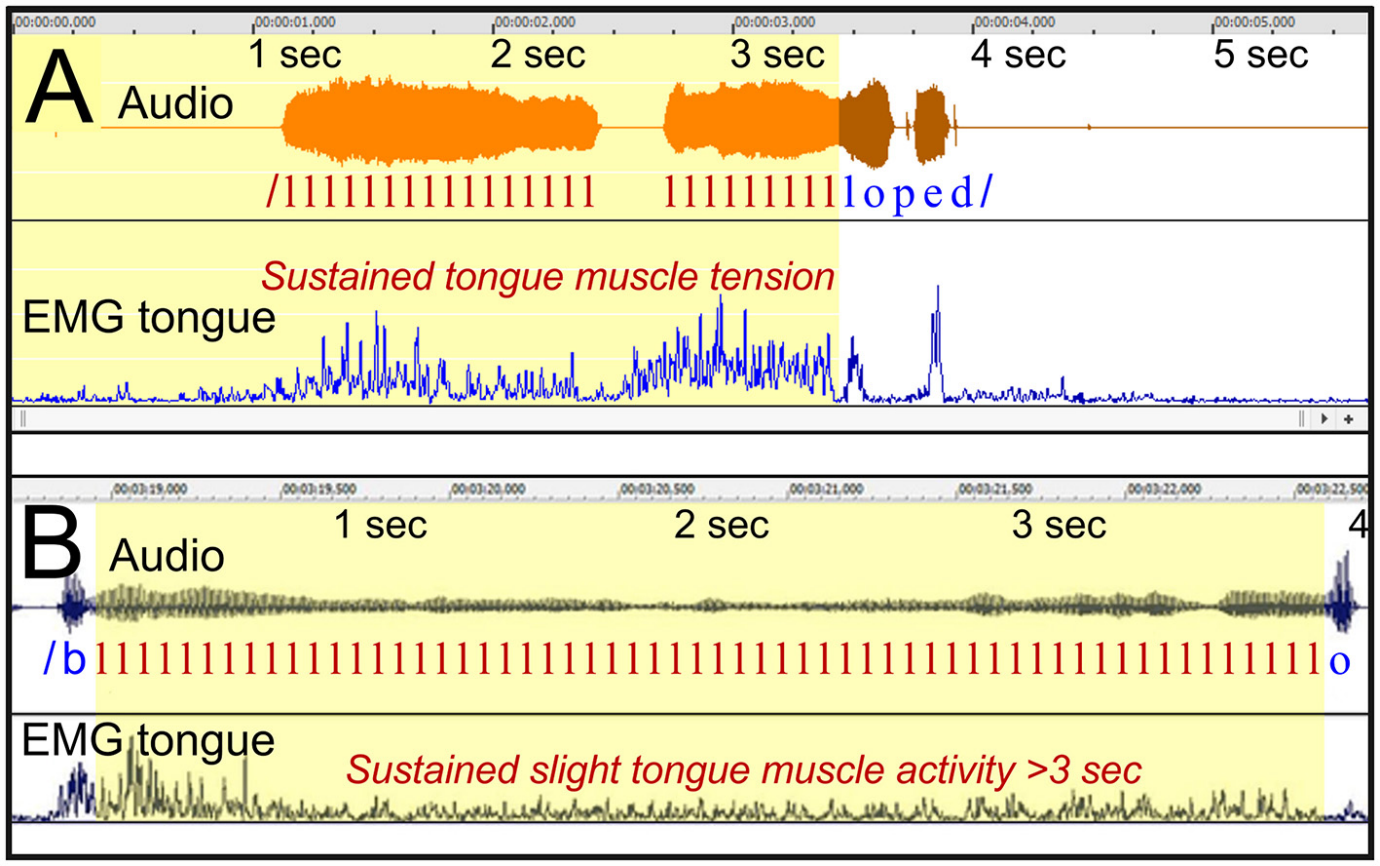
See Section 3.2.2.1. **(A)** Shows participant BC7 uttering the Swedish word “logoped” (pronounced as “loped”), with a 2.5 s prolongation of the initial /l/. The EMG of the tongue is moderately elevated during the prolongation. **(B)** Shows a similar prolongation of /l/ when participant BC11 is uttering the Swedish word “blockar.” The tongue EMG shows a relatively level during the prolongation.

*Participants*: Figure 11A: BC7, female. Figure 11B: BC11, male.

*Stuttering event*: Prolongation of /l/, in both examples.

*Tongue EMG*: Showing sustained elevated activity throughout the prolongations. In Figure 11A, the EMG level can be described as moderate, i.e., at approximately the same peak level as the activity during the fluent part of the utterance. In Figure 11B, the EMG level of the tongue is remarkably stable during the prolongation, at a low level.

*Interpretation*: In Figure 11A, the tongue showed stable sustained activity during the prolongation, with peaks at the same level as the functional contraction of the tongue during the later fluent production. This suggests that the level of contraction during the prolongation was within normal physiological limits, making dystonia an unlikely explanation in this case. Dystonia would be expected to result in contractions that are stronger than the level during fluent speech. Instead, the example is compatible with the interpretation of a “motor block.”

#### 3.2.3 Study C: video of the vocal folds + EGG

##### 3.2.3.1 Participant BC7: supraglottal closure of airway

*Figure*: 12.

*Participant*: BC7, female.

**Figure 12:**
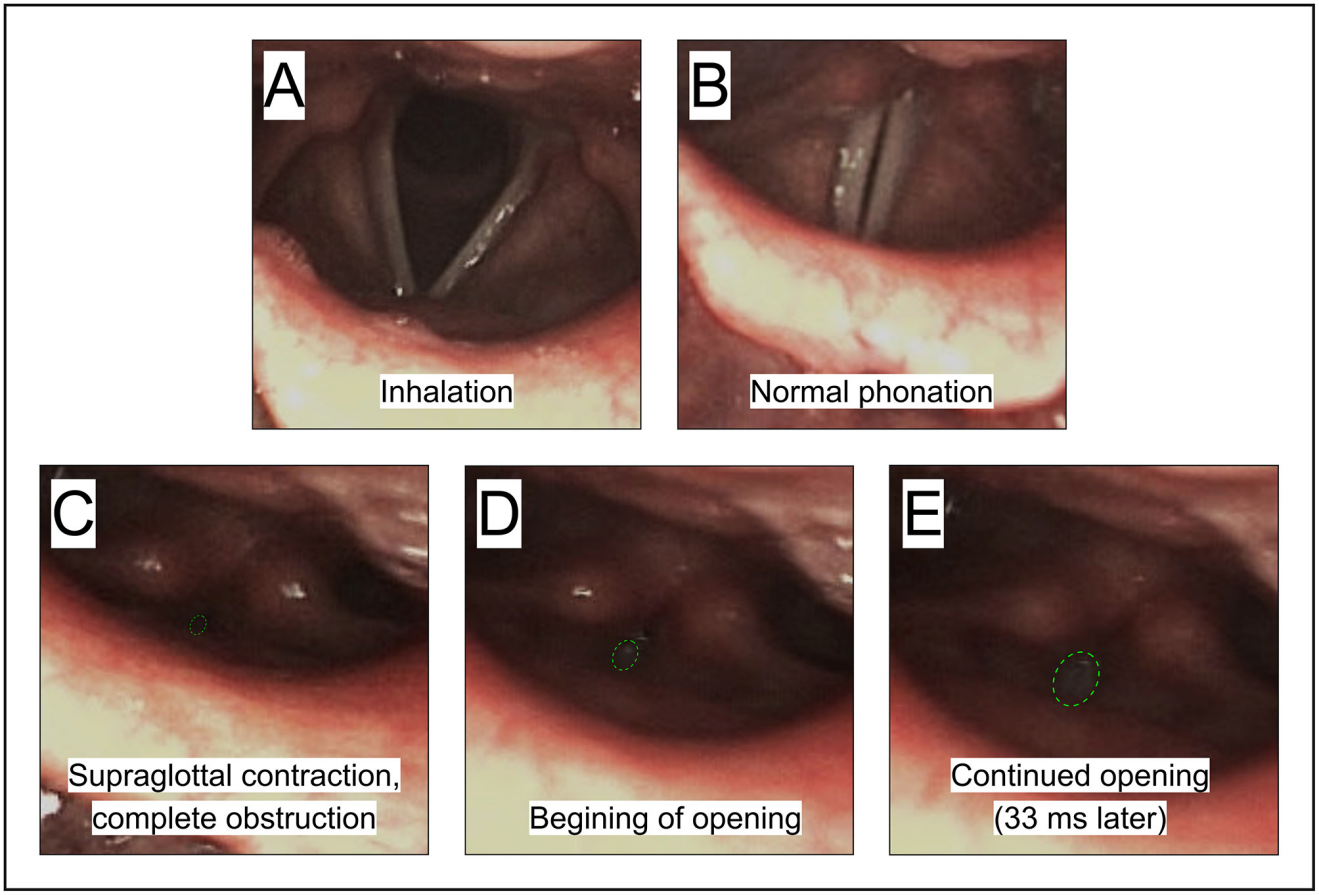
See Section 3.2.3.1. Participant BC7, female. **(A, B)** Exemplifies the vocal folds during normal inhalation and phonation, respectively. **(C)** Shows a complete blockage of airflow by the ventricular folds and adjacent structures when trying to initiate phonation to say “ena.” **(D, E)** Show the gradual opening before the closure is resolved.

*Stuttering event*: Figure 12 shows an attempt to say “var ena.” The participant was temporarily unable to initiate /e/ in “ena,” resulting in a word repetition: “var var ena.”

*Supraglottal constriction*: The inability to initiate the vowel was linked to a complete closure of the vocal tract immediately above the vocal folds, by the ventricular folds in combination with the lower part of the epiglottis.^3^ See Figures 12C–E for sequence. This example is similar to what was shown by Ramig (2004).

*Summary*: This is an example of blockage of airflow, occurring when trying to initiate phonation. The main point of blockage in this case is at the level of the ventricular folds.

##### 3.2.3.2 Participant BC6 “Katarina”: vocal fold tremor and pharyngeal contractions

*Figure*: 13.

*Participant*: BC6, female.

**Figure 13:**
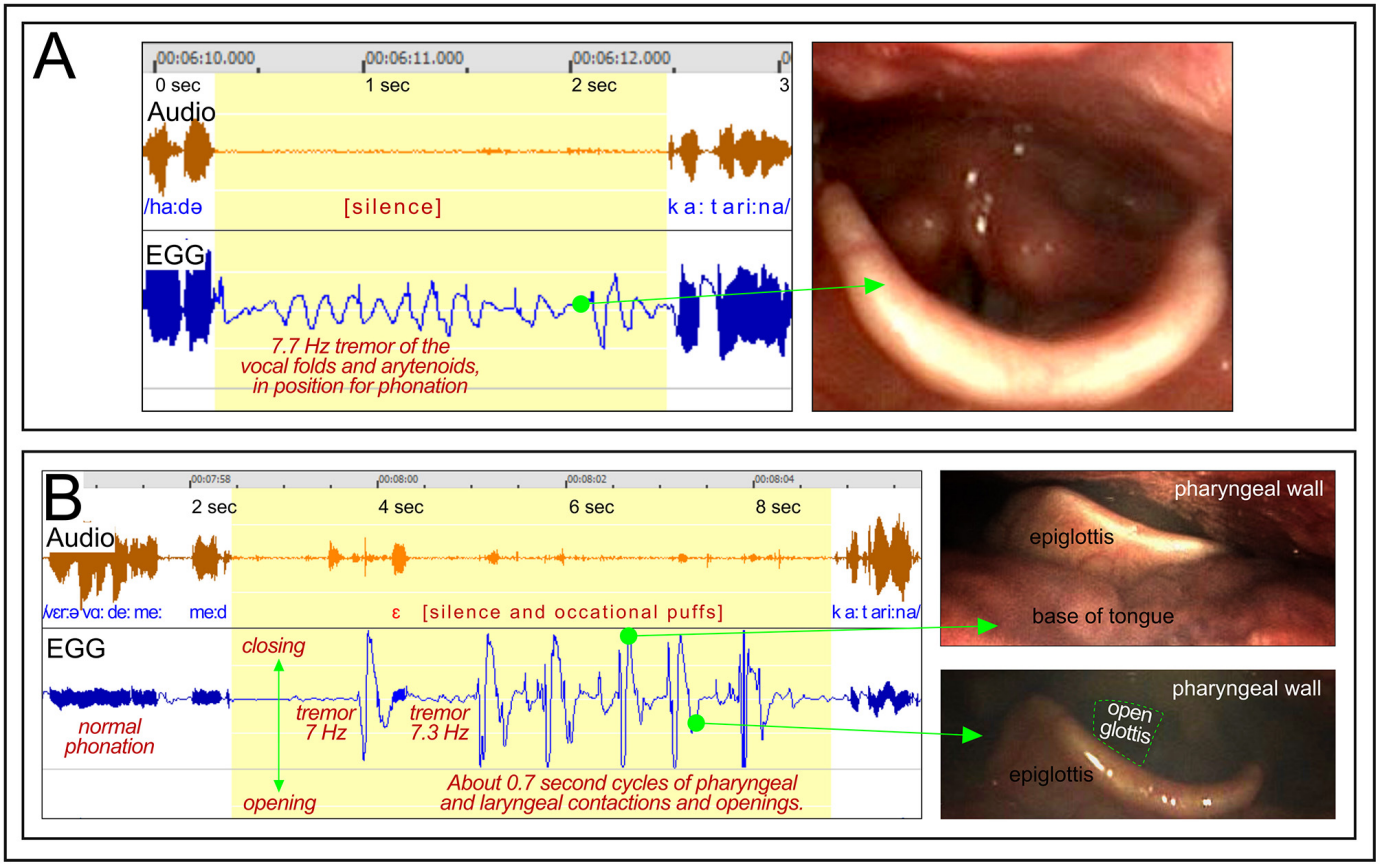
See Section 3.2.3.3. Participant BC6. The panels show two stuttering events occurring when attempting to say “Katarina.” **(A)** Shows stuttering with mild 7.7 Hz tremor of the vocal folds when in approximate position for phonation. **(B)** Shows stuttering with a combination of mild tremor and high-amplitude rhythmic contractions in the pharyngeal region.

*Stuttering events*: Figures 13A, B show stuttering events with a silent period before the word “Katarina.” In order to say this word, the vocal folds need to be separated for the /k/ but closed for phonation for the following /a/, and then separated again for the /t/.

*Dysfunctional laryngeal behavior*. During the stuttering event shown in Figure 13A, the vocal folds appear to remain in position for phonation. The video shows a slight trembling of the arytenoid cartilages, which in the EGG waveform is displayed as a mild 7.7 Hz tremor. The occurrence of the tremor indicates that the motor control of the laryngeal muscles is temporarily dysregulated. The stuttering event in Figure 13B also shows this type of mild tremor, but in particular repeated cyclic closures and openings of the pharyngeal region, with an interval of about 0.7 s. What is happening there is not clear, but the photos to the right in Figure 13B show that the tongue is retracted during the closed phase, and that the vocal folds are abducted during the open phase.

*Interpretation*: These two events suggest that the motor control of the larynx may enter a dysregulated state before challenging motor demands, such as the production of the word /Katarina/. The word requires a rapid series of exact openings and closings of the vocal folds. The symptoms are consistent with a “motor block.” Similarly to the motor blocks in FOG, these stuttering blocks occurred when a challenging shift of motor patterns was required—in this case a multisyllabic word beginning with a CVCV sequence, with two voiceless plosives. This implies a need to rapidly initiate and terminate phonation. It is known from previous studies that stuttered disruptions tend to be more frequent in longer words, and, in adults, in words beginning with consonants (Bloodstein et al., 2021).

##### 3.2.3.3 Participant BC11 open vocal fold oscillations

*Figure*: 14A.

*Participant*: BC11, male.

**Figure 14:**
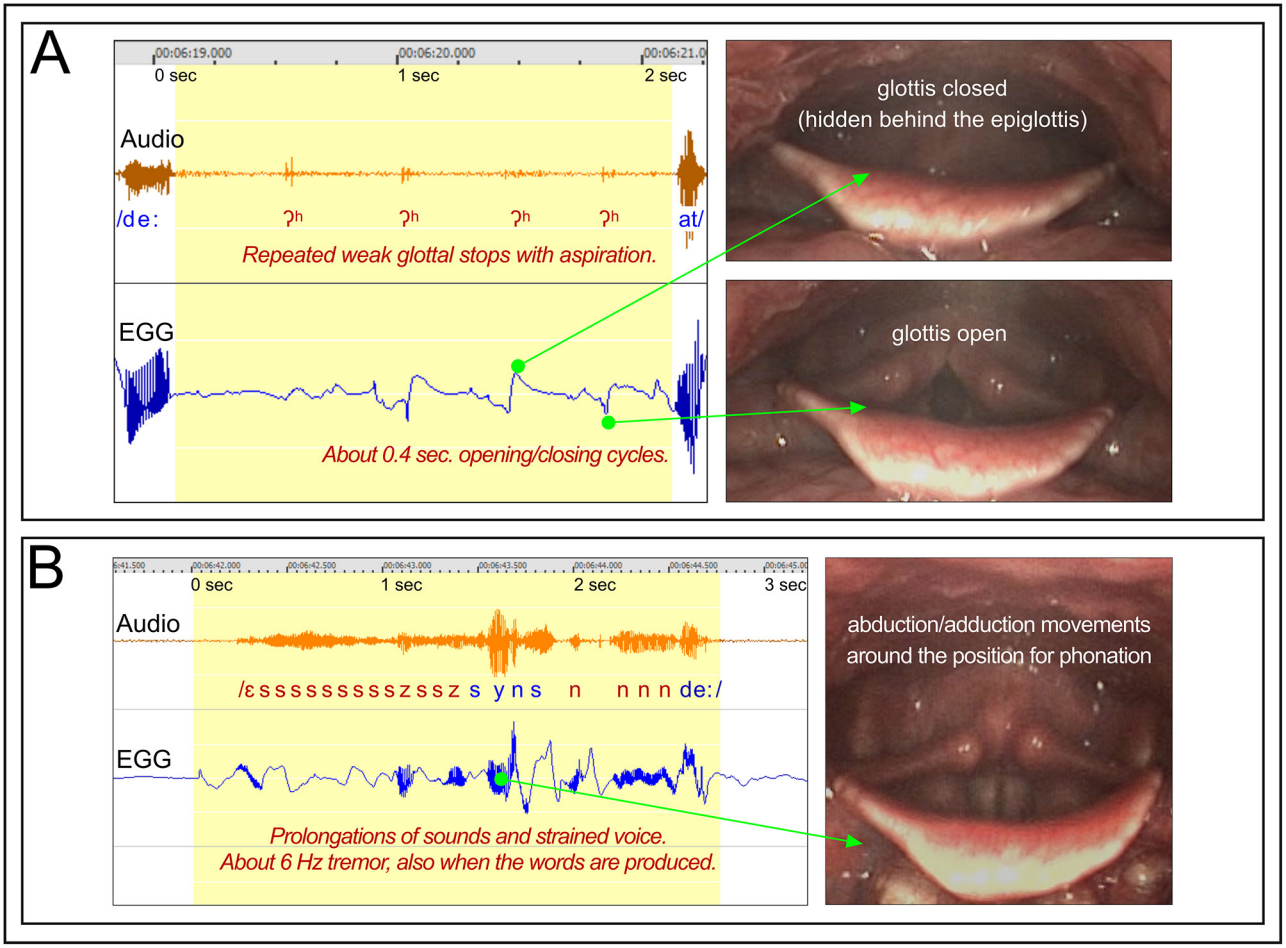
Participant BC11. **(A)** See Section 3.2.3.3. Shows a silent period before the word “att,” with opening and closings of the vocal folds with about 0.4 s intervals, resulting in weak glottal stops with aspiration. **(B)** See Section 3.2.3.4. Shows stuttering on the utterance “syns det,” with prolongations and tremor.

*Stuttering event*: Figure 14A shows a 2 s silent period before the word “att.” Within the silent period, four weak voiceless glottal stops with aspiration can be heard, with about 0.4 s intervals, indication opening and closing of the vocal folds.

*EGG and video*: The EGG waveform confirms an opening/closing cycle of about 0.4 s. The endoscopic video shows oscillatory movements of the vocal folds, with the 0.4 s cycle, from open to closed glottis.^4^

*Summary*: In order to produce the word “att,” the vocal folds must stay in the position for phonation long enough to produce the /a/. In this case the phonation was precluded by the opening and closing movements of the vocal folds.

##### 3.2.3.4 Participant BC11 open vocal fold oscillations

*Figure*: 14B.

*Participant*: BC11, male.

*Stuttering events*: Figure 14B, shows the utterance “syns det,” with prolongation on both /s/ and /d/. Because /d/ is a plosive, the prolongation became /n/, with the tongue position for /d/ but with nasalization. In addition, the whole utterance is affected by an audible 6 Hz tremor.

*EGG and video*: The EGG waveform shows an irregular tremor at ~6 Hz, which is reflected in the endoscopic video, with abduction/adduction movements of the vocal fold.

*Summary*: This utterance is unusual as the tremor continues also when the words actually are produced, which is heard in the audio recording. Typically, tremor in stuttering is limited to the stuttering event.

## 4 Discussion

The main results of this initial and exploratory study will be summarized and discussed below.

### 4.1 Stuttering in relation to known movement disorders

In Section 1.4, the characteristics of dystonia, tremor, and motor blocks were reviewed. The purpose of that review was to provide a framework and terminology for comparison with the symptoms and characteristics of stuttering.

First, dystonia and stuttering differ in that excessive tension is a core aspect of dystonia, but not in stuttering. In contrast, the tension in stuttering events can vary from being lower than normal, normal, to being very high. However, it cannot be excluded that aspects of dystonia are involved in stuttering, in particular in advanced stages, for example resulting in supraglottal contractions blocking the airflow.

Second, though tremor is common in stuttering, it does not appear to be a primary aspect, as it seems to be a symptom that develops, in some cases of advanced stuttering, often at school age (Denny and Smith, 1992; Kelly et al., 1995; Walsh and Smith, 2013). The analysis in the present study suggests that tremor tend to emerge from a static position, and may reflect attempts of the speaker to break a fixed posture.

Third, the concept of “motor blocks” by Giladi et al. (1992) appears to be compatible with most of the core symptoms and the subjective experiences related to stuttering. The stop in movement is not caused by a sudden spasm or cramp, with clearly excessive muscular tension, but rather by the system entering a temporarily unresponsive state, where the current motor position is maintained. In this way, a motor block can display both negative and positive symptoms.

In summary, the characteristics of motor block disorders, as exemplified by FOG (e.g., Nutt et al., 2011), share many of the characteristics of stuttering discussed in this article:

1. Affecting well-learned motor sequences, that typically are performed with a high degree of automaticity.
2. The motor control for specific muscles becomes temporarily unresponsive.
3. The motor symptoms are both negative and positive, and the muscular tension is typically within normal levels.
4. The subjective experience of the episodes is loss of control, and a feeling of being stuck.
5. Tremor can be shown and are suspected to emerge as a result of the individuals attempt to resolve the fixed position.
6. The episodes are commonly brief, a couple of seconds, but may exceed 30 s.
7. The episodes typically appear when starting a sequence, or when the sequence requires more challenging changes of the movements.
8. The risk for episodes can be temporarily reduced by means of various “sensory tricks” or manipulation of attention. For example, to move/talk to the pace of a rhythm; to have external signals to aim at, for example stripes on the floor or markers for the rhythm in a text.
9. The emotional and cognitive condition can have striking effect both on FOG and stuttering, both alleviating and aggravating the symptoms, temporarily.
10. Research on the brain mechanisms of stuttering points toward the putamen, i.e., the motor part of the striatum within the basal ganglia, as playing a main role.

### 4.2 Negative motor signs

Stromsta and Fibiger (1980) reported missing or weak anticipatory coarticulation in repeated segments in part-word repetitions, which they interpreted as a result of impaired planning of coarticulation. Instead, it is here proposed that these symptoms represent *negative motor signs*—that is, speech is interrupted as a result of missing or insufficient activation of muscles needed for the speech process. Such signs have been observed in at least 3 of 11 participants in the present study, as described in Section 3.1. Interestingly, these three were also the three persons showing the lowest ratio between the physical concomitants scores and the percentage of stuttered syllables. In other words, these three tend to show relatively low levels of muscular activation in relation to their frequency of stuttering, which aligns with the proposal of stuttering as a negative motor sign in these cases.

However, one of these three participants (A5) also showed positive motor signs related to stuttering. The clearest example is shown in Figure 10, with strong tremor of the lip muscles, resulting in fast part-syllable repetition. In addition, in Figure 5 he shows sustained OO muscle activity in parallel with missing OO activity. This is an example of a combination of negative and positive motor signs. This symptomatology is consistent with a “motor block.”

### 4.3 Tremor

Van Riper (1963, p. 330) was of the opinion that tremor in stuttering tend to appear when an articulatory posture becomes tense. This is supported by some of the examples in the present study, with tremor emerging from static muscular contraction. The clearest example is in Figure 5, with onset of tremor after 2.5 s of fixed posture and audible prolongation with lip closure. However, in other examples, tremor can start after just a few tenths of a second of fixed posture, as in Figures 6, 9, 10. An interpretation of these examples is that the tremor can emerge as a result of the intention of the speaker to continue to talk, thereby interfering with a temporarily fixed motor state, as noted by Gironell and Kulisevsky (2009) regarding tremor in dystonia. The tremor might occur as kind of “tug of war” within the motor system, with conflicting signals from the basal ganglia and the cortex. The dynamics of the tremor are likely to also involve the cerebellum, which has the function of trying to correct ongoing movements in relation to the output of the cortical motor neurons (Purves et al., 2019, p. 403). It appears possible that the tremor primarily is a cerebellar effect, trying to resolve the situation but overcompensating in both directions.

One previously unreported effect of tremor in stuttering is that fast repetitions of sounds or syllable fragments can result directly from the tremor. This happens when the tremor results in rapid opening and closing of the airway at some level of the vocal tract. Figure 9 shows one example, during the period between 0.8 and 1.6 s in the graph. What happens here is that the lip opening muscle, DLI, shows a tremor of exactly the double frequency compared to the lip closing muscle, OO. The OO is stronger than the DLI, so that the lips close when the two muscles co-contract. However, because DLI have peaks when the OO is inactive, this will result in opening of the lips. Repetitions of brief voiced sounds can also be the result of laryngeal tremor resulting in an abductory/adductory movement with the tremor frequency. The voiced sound is heard when the vocal folds briefly meet in a position for phonation. Figure 14A, shows this type of mechanism resulting in repeated glottal stops. It is our impression that most repetitions in stuttering are not a direct effect of tremor, but that this phenomenon is still relatively common.

A “tremor index,” presented in Section 3.2.1.1, was calculated as the ratio between the spectral power of the EMG in typical tremor frequencies (7–20 Hz) and the spectral power in frequencies typical for normal speech (1–5 Hz), during stuttering events. The tremor index showed a correlation of 0.77 with the SSI physical concomitants score, see Figure 4. This suggests a strong relationship between tremor and physical concomitants. However, there seems to be a significant amount of individual variation, as participant A10 in Figure 4 showed high physical concomitants score but a relatively low tremor index.

### 4.4 Reliability of the results and limitations of the study

The present study is best viewed as foundational, aiming to: (1) develop multimodal methodologies for analyzing speech motor events, (2) conduct a detailed exploratory analysis of initial data, and (3) compare stuttering symptomatology with established neurological movement disorders. We prioritized characterizing the heterogeneity of speech disruptions, which varied across several key dimensions:

1. Traditional clinical classifications
2. (repetitions, prolongations, blocks).
3. Repetition rates and segment lengths.
4. Presence/absence of tremor, with differing frequency and dynamics.
5. Presence/absence of static muscular contraction.
6. Missing muscular activity in repeated segments.
7. Locus of anomalies (primarily lips, tongue, or larynx).

Given this complexity, systematic quantification of the prevalence of specific behaviors in a larger cohort remains challenging. Therefore, initial qualitative exploration is critical to establish criteria for future quantitative studies, including standardized event classification and interrater reliability assessments. While the current design does not permit formal reliability metrics, we provide numerous high-resolution examples to enable reader evaluation and facilitate replication.

### 4.5 Alternative theories?

#### 4.5.1 Is stuttering a result of active action inhibition from the frontal cortex?

A theory of stuttering that has emerged during the last decade is that the disruptions are the result of active inhibition of speech output. The theory is based on the proposal of Aron et al. (2004) and Hannah and Aron (2021), that regions within the right prefrontal cortex are central for a global response inhibition system. These cortical regions would send signals to the basal ganglia in order to globally stop ongoing motor actions, or prevent motor actions. This action inhibition framework has been applied to stuttering in different formulations by Neef et al. (2016, 2018), Arenas (2017), Hannah and Aron (2021), Jackson et al. (2022), and Orpella et al. (2024).

So, is active global speech motor inhibition from the frontal lobe, via the basal ganglia, consistent with the results of the present study? First, if such a mechanism would inhibit speech output, it could be expected that all speech effectors are inhibited simultaneously. This is not the case in our studies. For example, Figures 1–3 show negative motor signs affecting the articulators, but the phonation is unaffected until about 100–300 ms after the expected articulatory activity. Similarly, Figures 5 and 11 show audible prolongations with normal phonation. In these examples, the lips or the tongue are in a fixed position, while the phonation is unaffected. Failure of specific speech muscles to get activated, while others respond normally, rather suggests that the problem occurs at a low level within the motor system, not being related to global commands. Second, it is not clear why a global inhibition mechanism would result in prolonged loss of volitional control and the experience of being “stuck.”

However, it is still possible that this type of inhibitory mechanisms can have a modulating effect on the symptoms of stuttering, even though it is not a core mechanism. Such a mechanism may explain much of the contextual variability of stuttering, i.e., influencing when stuttered disruptions will occur or not. This is in line with statements by Arenas (2017) and Orpella et al. (2024).

#### 4.5.2 Are stuttering disruptions the result of motor variability?

Speech motor control theories of stuttering focus on possible deficits in the ability to produce exact speech movements. Smith et al. (2012) found that children who stutter tend to exhibit a higher degree of variability of oral movements during production of fluent utterances, compared to other children. Civier et al. (2010) proposed a model in which stuttering arises from an overreliance on auditory feedback control, leading to articulatory errors. It was further assumed that when such errors grow large enough, they can cause the motor system to interrupt and restart the current syllable. However, the predictions of this model do not seem to match what we have observed during stuttering events. While we observed that absent muscular activity leads to interruptions and restarts (Section 3.1), this specific absence of single muscles cannot be easily attributed to speech motor control instability or excessive reliance on auditory feedback. Moreover, motor variability accounts do not seem to be able to explain blocks occurring before speech initiation.

### 4.6 Proposal of brain mechanism for “motor blocks” in stuttering

If motor blocks do constitute the core of stuttering, what brain mechanisms might underlie this symptom? Examining research on established motor block disorders—particularly freezing of gait (FOG) in Parkinson's disease—may provide insights.

It is here proposed that the primary mechanism triggering motor blocks in stuttering is a transient functional decoupling between executive/motor *cortical* networks and *basal ganglia* motor circuits. The risk for decoupling increases with white matter impairments and with pathophysiological differences within the basal ganglia system, affecting its responsiveness. The principles of this model were developed as an explanation for FOG, by Shine et al. (2013). They found that a frontal cortical network lost connectivity with the basal ganglia network before FOG episodes, and restored the connectivity after the episode. This model offers a plausible explanation for the perceived loss of control in both FOG and stuttering. It also has the potential to explain many of the phenomena related to the variability of stuttering, such as the complex effects of attention and emotions.

The idea that stuttering is related to the basal ganglia system emerged already in the 1920s in Central Europe (summarized in Alm, 2005). More recently, this possibility has been put forward by Maguire et al. (2002), Alm (2004), Civier et al. (2013), Chang and Guenther (2020), and others. These publications suggest a dysfunction of the basal ganglia structures and/or the reciprocal connections between the cortex and the basal ganglia. Interestingly, Lu et al. (2010) reported elevated positive effective connectivity *from* the putamen *to* cortical regions (via the thalamus). That is, a stronger than normal “bottom-up” influence. A stronger than normal effective connectivity from the putamen to these cortical regions may imply a reduced ability to volitionally control the activity of the basal ganglia, with an increased risk for communication difficulties between the two levels. Differences in *functional* connectivity (not showing direction) between basal ganglia nuclei and cortical regions have been reported by Chang and Zhu (2013), Chang et al. (2016), and others.

### 4.7 Conclusions

The results of this initial and exploratory study support the idea that stuttering events can be associated with *negative* motor signs—that is, missing or insufficient activation of the speech muscles needed. At the same time, the data have also shown *positive* motor signs, in the form of sustained contractions during fixed postures, and in the form of tremor. The sustained contractions typically involve moderate levels of muscular tension, while high levels of tension are typically associated with tremor. Tremor appears to emerge from sustained contraction, possibly due to attempts by the speaker to resolve a fixed posture.

The main preliminary conclusion, based on a review of the literature combined with analysis of the new data, is that the core symptoms of stuttering appear to be compatible with the concept of “motor blocks” (as defined by Giladi et al., 1992). Such motor blocks can result in a combination of negative and positive motor signs. In addition, some symptoms may present features typical of dystonia. Lastly, a form of dystonic tremor may reflect efforts to continue the speech sequence, resulting in physical concomitants. It is proposed that the core motor blocks stem from transient decoupling between cortical and basal ganglia networks.

## Data Availability

The datasets presented in this article are not readily available because the dataset include audio of speech and video of the face, which make participants identifiable. This data will not be shared. Parts of the data, not including identifiable information, may be shared on motivated request. Requests to access the datasets should be directed to per.alm@uu.se.

## Footnotes

^1^“Gluency” is a word invented by Van Riper, as a mix of glue and fluency, to describe the stickiness of stuttered speech.

^2^In the initial tongue EMG studies, EasyCap E271 circular flat 4-mm Ag/AgCl electrodes were attached to the tongue using biocompatible cyanoacrylate tissue adhesive (GluStitch PeriAcryl 90HV), but this method was discontinued in favor of disposable electrodes due to convenience and signal quality.

^3^For details on supraglottal laryngeal mechanics, see discussion in Lindblom (2009).

^4^When the glottis was closed it became hidden by the epiglottis in the video recording. However, the approximate position of the vocal folds could be determined by the visible positions of the arytenoid cartilages.
